# Brief cycling intervals incrementally increase the number of hematopoietic stem and progenitor cells in human peripheral blood

**DOI:** 10.3389/fphys.2024.1327269

**Published:** 2024-07-30

**Authors:** Fendi Pradana, Tarondeep Nijjar, Phoebe A. Cox, Paul T. Morgan, Tim Podlogar, Samuel J. E. Lucas, Mark T. Drayson, Francesca A. M. Kinsella, Alex J. Wadley

**Affiliations:** ^1^ School of Sport, Exercise, and Rehabilitation Sciences, University of Birmingham, Birmingham, United Kingdom; ^2^ Nutrition Study Program, Faculty of Public Health, Tadulako University, Palu, Indonesia; ^3^ Department of Sport and Exercise Sciences, Manchester Metropolitan University, Manchester, United Kingdom; ^4^ Clinical Immunology Service, University of Birmingham, Birmingham, United Kingdom; ^5^ Institute of Immunology and Immunotherapy, University of Birmingham, Birmingham, United Kingdom; ^6^ Centre for Clinical Haematology, Queen Elizabeth Hospital, Birmingham, United Kingdom

**Keywords:** peripheral blood stem cell donation, exercise, interval cycling, HSPC, natural killer cell, transplantation

## Abstract

**Introduction:**

Peripheral blood stem cell (PBSC) donation is the primary procedure used to collect hematopoietic stem and progenitor cells (HSPCs) for hematopoietic stem cell transplantation. Single bouts of exercise transiently enrich peripheral blood with HSPCs and cytolytic natural killer cells (CD56^dim^), which are important in preventing post-transplant complications. To provide a rationale to investigate the utility of exercise in a PBSC donation setting (≈3 h), this study aimed to establish whether interval cycling increased peripheral blood HSPC and CD56^dim^ concentrations to a greater degree than continuous cycling.

**Methods:**

In a randomised crossover study design, eleven males (mean ± SD: age 25 ± 7 years) undertook bouts of moderate intensity continuous exercise [MICE, 30 min, 65%–70% maximum heart rate (HR_max_)], high-volume high intensity interval exercise (HV-HIIE, 4 × 4 min, 80%–85% HR_max_) and low-volume HIIE (LV-HIIE, 4 × 2 min, 90%–95% HR_max_). The cumulative impact of each interval on circulating HSPC (CD34^+^CD45^dim^SSC^low^) and CD56^dim^ concentrations (cells/µL), and the bone marrow homing potential of HSPCs (expression of CXCR-4 and VLA-4) were determined.

**Results:**

There was an increase in HSPC concentration after two intervals of LV-HIIE (Rest: 1.84 ± 1.55 vs. Interval 2: 2.94 ± 1.34, *P* = 0.01) and three intervals of HV-HIIE only (Rest: 2.05 ± 0.86 vs. Interval 3: 2.51 ± 1.05, *P* = 0.04). The concentration of all leukocyte subsets increased after each trial, with this greatest for CD56^dim^ NK cells, and in HIIE vs. MICE (LV-HIIE: 4.77 ± 2.82, HV-HIIE: 4.65 ± 2.06, MICE: 2.44 ± 0.77, *P* < 0.0001). These patterns were observed for concentration, not frequency of CXCR-4^+^ and VLA-4^+^ HSPCs, which was unaltered. There was a marginal decrease in VLA-4, but not CXCR-4 expression on exercise-mobilised HSPCs after all trials (*P* < 0.0001).

**Discussion:**

The results of the present study indicate that HIIE caused a more marked increase in HSPC and CD56^dim^ NK cell concentrations than MICE, with mobilised HSPCs maintaining their bone marrow homing phenotype. LV-HIIE evoked an increase in HSPC concentration after just 2 × 2-minute intervals. The feasibility and clinical utility of interval cycling in a PBSC donation context should therefore be evaluated.

## 1 Introduction

Single bouts of exercise evoke rapid increases in the concentration of various subsets of immune cells in peripheral blood (Walsh et al., 2011; Simpson et al., 2015). This is part of a dynamic response that transiently enriches blood with cells that govern skeletal muscle repair and immunosurveillance in exercise recovery (Simpson et al., 2021). Notably, the number of effector immune cells (e.g., cytolytic natural killer (NK) cells (CD56^dim^) and cytotoxic T cells) increase between ∼4 and 10-fold during exercise, whereas pluripotent cells such as haematopoietic stem and progenitor cells (HSPCs) increase by ∼2–3 fold (Bonsignore et al., 2010; Krüger et al., 2015). There has subsequently been an emerging clinical interest in the potential of exercise to enrich the immune cell fraction or “graft” during peripheral blood stem cell (PBSC) donations (Porrata et al., 2004; Graff et al., 2018), which are used to treat conditions such as multiple myeloma and lymphoma (Porrata et al., 2004; Giralt et al., 2014; Carreras et al., 2018) and rare blood, autoimmune and congenital metabolic diseases (Henig and Zuckerman, 2014).

Exceeding the HSPC collection threshold (>2 × 10^6^ cells/kg) is critical for patients undergoing PBSC donations (termed “autologous donors”) (Sezer et al., 2000). In response to standard mobilisation therapy using granulocyte colony stimulating factor (G-CSF), many autologous donors are classified as “poor mobilisers” (≈40%) due to prior treatments damaging the bone marrow (e.g., myeloablative chemotherapy). This results in delayed and compromised engraftment of HSPCs, repeated hospital visits, inability to deliver further treatments and poorer health outcomes. HSPC mobilisation failure is much less common (≈5%) in healthy human leukocyte antigen (HLA)-matched donors (termed “allogenic donors”). However, the collection of effector immune cells such as CD56^dim^ cells alongside HSPCs is of paramount importance to reduce this risk of disease relapse in the recipient after transplant (Maggs et al., 2017). Although the magnitude (∼4 cells/µL) of the HSPC response to exercise falls short of the collection threshold needed to begin apheresis (>10 cells/µL), a forward-thinking hypothesis has been that exercise in combination with G-CSF may help to achieve the HSPC dosing threshold required for successful engraftment and expedite the PBSC donation process (Baker et al., 2017). Furthermore, enrichment of the graft with CD56^dim^ and other effector immune cells may offset the risk of post-transplant viral infections and graft-versus-host disease (GvHD) by priming adaptive immune responses in the recipient (Porrata et al., 2004).

In addition to the number of harvested HSPCs, the engraftment phenotype of these cells is critical for predicting clinical endpoints following transplant, and some evidence indicates that exercise may modulate this. For example, pluripotent hematopoietic stem cells (HSCs: CD34^+^ CD38^-^) are known to predict trilineage engraftment success following autologous transplants (Hénon et al., 2001). Furthermore, higher expression of bone marrow homing receptors C-X-C chemokine receptor type 4 (CXCR-4) (Felker et al., 2022) and Very Late Antigen-4 (VLA-4) (Potocnik et al., 2000) on HSPCs promotes their successful engraftment in animal models and some human data indicate an association between CXCR-4 expression and engraftment success (Asfour et al., 2017). Exercise can mobilise CXCR-4^+^ HSPCs (+500 cells/mL) (Taylor et al., 2021) and CD34^+^ CD38^-^ (+3,000 cells/mL) (Baker et al., 2017; Nederveen et al., 2020); however, this largely reflects the typical leukocytosis associated with exercise, and the impact of different types of exercise on the cell surface expression of these receptors has not been reported. The complementary ligands to these receptors C-X-C motif chemokine 12 (CXCL-12) and vascular cellular adhesion molecule-1 (VCAM-1) increase in the circulation immediately after exercise in humans (Brevetti et al., 2001; Schmid et al., 2021a) and the expression of CXCL-12 is upregulated in skeletal muscle within 15 min of exercise onset in mice (Emmons et al., 2016a). These changes may provide chemoattractant cues for HSPCs to egress from vascular walls or the bone marrow into peripheral blood (Brzoska et al., 2012), thus making them available for harvest during PBSC donations.

Before addressing the feasibility of translating this concept into a clinical setting, the optimal dose of exercise to maximise HSPC and CD56^dim^ NK cell concentrations needs to first be established. During exercise, HSPCs and CD56^dim^ NK cells are rapidly mobilised from marginal pools within the circulation and tissues by shear stress and beta 2 (β2) adrenergic dependent mechanisms (Agha et al., 2018), with resting cell numbers restored within 5 (Schmid et al., 2021b) and 20 (Barra et al., 2017) min, respectively. Although many studies have reported increases in HSPCs after bouts of steady-state exercise lasting 30–45 min (Emmons et al., 2016a; Baker et al., 2017; Agha et al., 2018), this duration doesn’t align with the timeline of a PBSC donation session, which often lasts 3–4 h and often extends to multiple days. Given the known mechanisms underpinning HSPC mobilisation and rapid margination in response exercise, adopting periods of rest between intervals of high intensity exercise might therefore be a feasible approach. High intensity interval exercise (HIIE) of both high volume (5 × 3-minute cycling intervals at 90% peak power) and low volume (6 × 20 s “all-out” cycling sprints) (O’Carroll et al., 2019) have been reported to increase HSPC concentrations after the final interval relative to rest. However, one study evaluating moderate volume HIIE (≈10 min of total intervals at 90% of maximal heart rate) reported no change (Nederveen et al., 2020). In contrast, there are consistent data reporting increases in circulating CD56^dim^ NK cell concentrations after HIIE, ranging from 10 to 15 intervals (60–90 s) at 85%–90% peak oxygen consumption (Turner et al., 2016; Barra et al., 2017; Arroyo et al., 2022). More prominent NK mobilisation compared to HSPCs is explained by higher β2 adrenergic receptor expression on the surface of NK cells.

Evidently, there is a complex interplay between the intensity, duration, and total work of intervals needed to maximise circulating HSPC concentrations, and this is not fully understood. Previous studies typically compare changes before and after the last interval of HIIE, rather than monitoring cumulative changes after each interval. Enumerating HSPCs using guidance set out by the International Society of Hematotherapy and Graft Engineering (ISHAGE) (Keeney et al., 1998) would facilitate such an approach. This single platform flow cytometric (SPFC) approach uses small volumes of whole blood (100 µL) to enable rapid and accurate quantification of peripheral blood HSPC concentrations. The advantage of using the SPFC approach compared to the previous exercise-based studies that have mostly used a double platform flow cytometry (DPFC) approach (i.e., flow cytometry coupled with automated haematology analysis) is that less blood is needed, processing times are shorter and both inter- and intra- laboratory variances are significantly less with SPFC than DPFC (Gratama et al., 2003).

Understanding how different volumes of HIIE impact the “quantity” and “quality” of HSPCs in peripheral blood over a suitable time course is a clear knowledge gap that needs addressing to evaluate the potential of exercise to work in combination with PBSC donations. For future adoption of this approach clinically, evaluation of HIIE protocols that are feasible for the donor are critical. Therefore, criteria outlined for utilising HIIE in clinical populations based on a percentage of maximal heart rate were used to guide protocol design (Weston et al., 2014; Taylor et al., 2019). Accordingly, the primary aim of this study was to compare changes in peripheral blood HSPC concentrations before and after each of four consecutive intervals of low volume HIIE (LV-HIIE) and high-volume HIIE (HV-HIIE) vs. a time-matched continuous cycling bout (control). Secondary aims included quantifying changes in CD56^dim^ NK cell concentrations, characterising the bone marrow homing potential of HSPCs mobilised with exercise, and contrasting single vs. double platform quantitative HSPC methods.

## 2 Materials and methods

### 2.1 Participants

Eleven healthy males were recruited into this study (mean ± SD: age 25 ± 7 years; body mass index: 25.7 ± 3.0 kg/m^2^). Participants underwent screening prior to enrolment and were deemed eligible if they were 18–45 year-old, not highly active (as defined by the General Practice Physical Activity Questionnaire (GPPAQ) (Ahmad et al., 2015), non-smokers, not currently taking medication, and had no previous history of cardiovascular, metabolic, neurological, or inflammatory diseases. All participants gave written informed consent before participating and the study was given favourable ethical opinion by the Science, Technology, Engineering and Mathematics ethical committee at the University of Birmingham (ERN_19-1574PA2).

### 2.2 Preliminary testing

Participants undertook four laboratory visits, including three randomised cycling trials at the School of Sport, Exercise and Rehabilitation Sciences at the University of Birmingham, conforming to the Declaration of Helsinki. On the first visit, participants initially rested for a period of 30 min followed by measurements of resting blood pressure (*Thuasne BP 3W1-A*, *Taipei*, *Taiwan*), height (*Seca Alpha*, *Hamburg*, *Germany*) and body mass (*Ohaus CD31*, *New Jersey*, *United States*). Participants then undertook a maximal power (Watt_max_) ramp test on an electromagnetically braked cycle ergometer (*Excalibur*, *Lode*, *Netherlands*). After a warm-up for 5 min at 50 W, the test started at 70 W and then 25 W increments were added every minute until volitional exhaustion. Heart rate (HR) was monitored continuously throughout (*H10*, *Polar Electro*, *Finland*). Participants were asked to maintain a cadence of ∼60 rotations/minute (RPM). Following a 15-minute rest period, participants were familiarised with the exercise protocols used in the three main trials. The intensity of these trials was based on a percentage of their maximal heart rate (HR_max_).

### 2.3 Experimental sessions

**FIGURE 1:**
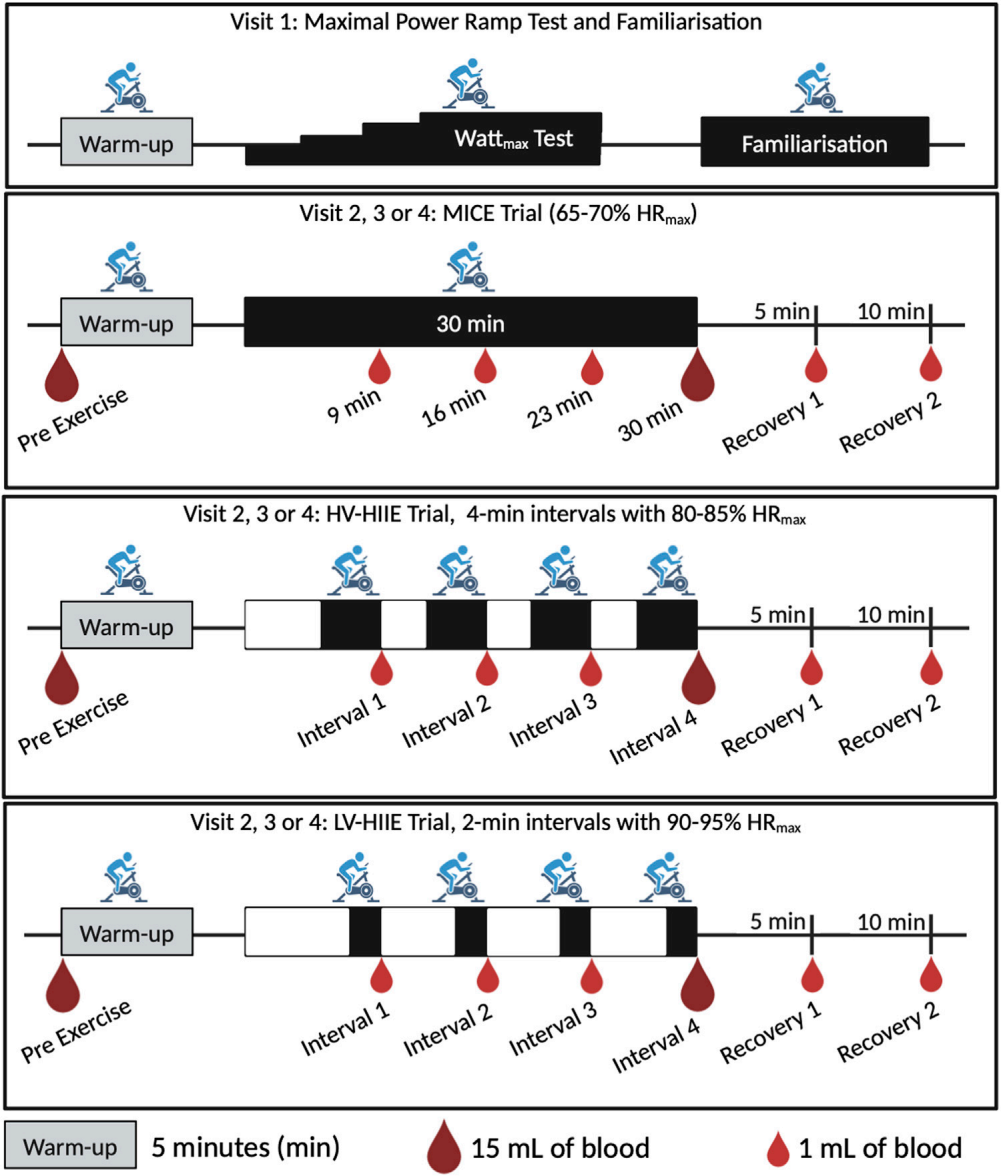
Study design illustrating a time axis of the warmup, intervals/steady state, and recovery cycling periods for the four laboratory visits and three randomised trials: MICE, Moderate intensity continuous exercise; HV-HIIE, High volume-high intensity interval exercise; LV-HIIE, Low volume-high intensity interval exercise. Blood sampling is indicated with small droplets (1 mL) for whole blood analysis, and large droplets (15 mL) for PBMC and plasma analyses. Created with BioRender.com.

The three main cycling trials were separated by at least 1 week and carried out at the same time of day (8:00–9.00 a.m. start time) and under stable climatic conditions (temperature: 19°C–20°C, humidity: 30%–55% and Barometric pressure: 1,000–1,050 hPa). Prior to each laboratory visit, participants were asked to undertake an overnight fast, refrain from vigorous exercise, and the consumption of caffeine and alcohol for 48 h. In the morning of each trial, participants were asked to drink 0.5 L of water within 4 h and 0.25 L within 15 min of commencing the trial. Questionnaires evaluating state and trait anxiety (Spielberger et al., 2017) and sleep efficiency (percentage of time asleep relative to the amount of time spent in bed) (Carney et al., 2012) were completed during a 30-minute period of rest where blood pressure and body mass were also measured. Following this a catheter (*Becton*, *Dickson* & *Company*, *Oxford*, *United Kingdom*) was inserted into the antecubital vein of the forearm and a baseline blood sample was taken (Rest). The catheter was kept patent through regular flushes with saline (0.9% NaCl, *Becton*, *Dickson* & *Company*, *Oxford*, *United Kingdom*). Each trial commenced with a 5-min warm-up at 50 W and then one of three randomised 30 min trials: 1) Moderate intensity continuous exercise (MICE) consisting of 30-minutes of cycling at 65%–70% HR_max_, 2) High volume-high intensity interval exercise (HV-HIIE) consisting of 4 × 4-minute cycling intervals at 80%–85% HR_max_ with 3 min of passive rest between each interval or 3) Low volume-high intensity interval exercise (LV-HIIE), consisting of 4 × 2-minute interval at 90%–95% HR_max_ with 5 min of passive rest between each interval. HR was monitored continuously alongside ratings of perceived exertion (RPE) (Robert and Brown, 1982) and the affective response using the Feeling Scale (Hardy and Rejeski, 2016) every minute. Total energy expenditure from each trial was estimated from power output using an equation proposed by Ettema and Lorås (2009). Six further blood samples were taken during each trial, one after each of the four intervals (and matched timepoints for MICE), and two samples in recovery (5–10 min following cycling completion). The seven samples were therefore named: Rest, Interval 1 (9 min), Interval 2 (16 min), Interval 3 (23 min), Interval 4 (30 min), Recovery 1 (35 min) and Recovery 2 (40 min). No fluid intake was permitted throughout the trials so as not to influence blood volume independent of the exercise itself; however, corrections were made (outlined below). A total of 35 mL of blood was taken during each trial, including 7 × 1-mL containing potassium ethylene-diamine-tetra-acetic acid (K_2_EDTA) vacutainers (*Greiner Bio-One*, *Frickenhausen*, *Germany*) for whole blood HSPC and complete blood counts (*Yumizen H500*, *Horiba*, *Kyoto*, *Japan*) at every timepoint. At Rest and after Interval 4 (30 min) only, 15-mL of blood was collected in K_2_EDTA vacutainers (*Becton*, *Dickson & Company*, *Oxford*, *United Kingdom*) for complete blood counts and then plasma and peripheral blood mononuclear cell (PBMC) isolation (Figure 1). The complete blood count was used to determine total white blood cell, neutrophil, monocyte, and lymphocyte concentrations (cells/µL), with haematocrit (L/L) and haemoglobin concentration (g/dL) also determined.

### 2.4 Blood processing

Plasma was obtained through centrifugation of 4 mL K_2_EDTA blood for 10 min at 1,525 × *g* at 4°C and stored at −80°C. PBMCs were isolated by gradient density centrifugation by first diluting 10 mL of whole blood with Dulbecco’s phosphate-buffered saline (*D-PBS*, *Thermo Fisher Scientific*, *Massachusetts*, *United States*) in a 1:1 ratio. Diluted blood was gently layered on the top of Histopaque-1077 separating medium (*Sigma Aldrich*, *Missouri*, *United States*) and centrifuged for 40 min, at 300 × *g* (break off) and 21°C. PBMCs were harvested by removing the PBMC interphase and washing three times with D-PBS before counting on a Cellometer 2,000 dual fluorescence cell counter (*Nexcelom Bioscience*, *Massachusetts*, *United States*). PBMCs were cryopreserved in freezing medium [RPMI (Roswell Park Memorial Institute) supplemented with 20% FBS (Fetal bovine serum) and 10% Dimethylsulfoxide] and stored in liquid nitrogen at the Human Biomaterials and Resource Centre at the University of Birmingham until analysis.

### 2.5 Flow cytometry data acquisition and analysis

Four colour flow cytometry analyses were undertaken using a CytoFlex-S flow cytometer (*Beckman Coulter*, *California*, *United States*): Analyses included determining: 1) concentration (cells/µL) of HSPCs in whole blood, 2) frequency of HSPCs, CD3^+^, CD56^dim^, and CD56^bright^ NK cells in the PBMC fraction and 3) frequency (%) and cell surface expression (Geometric Mean Fluorescence Intensity) of bone marrow homing receptor positive HSPCs (CXCR-4 and VLA-4) in the purified PBMC fraction. All antibodies used were purchased from BioLegend (*San Diego*, *CA*) or R&D Systems (*Minneapolis*, *United States*) and data were analysed with CytExpert v2.5 Software (*Beckman Coulter*, *California*, *United States*). Presentation of gating strategies was carried out using FlowJo™ v10.9 Software (*Becton*, *Dickson & Company*, *Ashland*, *United States*). Gates were formed using fluorescence minus one (FMO) controls, and compensation was applied for each trial/participant using single stained controls. Dead cells were excluded using a viability exclusion dye.

#### 2.5.1 Single platform flow cytometry

**FIGURE 2:**
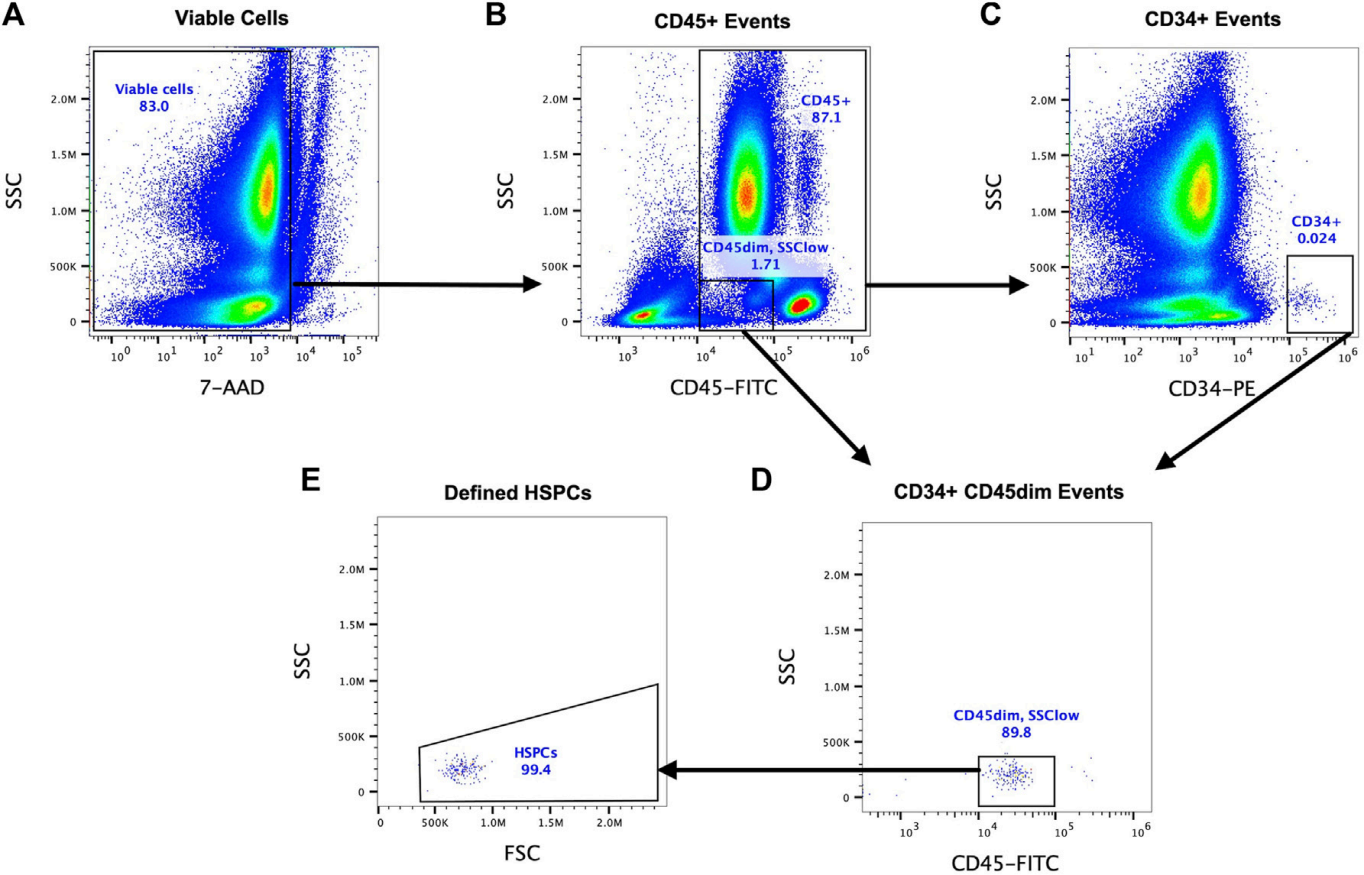
A Boolean gating strategy was used to enumerate HSPCs following guidelines validated by the International Society of Hematotherapy and Graft Engineering (ISHAGE) using whole blood (single platform, presented herein) or PBMCs (double platform). CD34^+^ cells were first identified by sequential gating of viable cells using 7-AAD exclusion **(A)**, CD45^+^ events **(B)** and then CD34^+^ events **(C)**. A gate on plot B defining viable cells with low to moderate (dim) expression of CD45 and low side scatter properties was combined with CD34^+^ cells using Boolean gating **(D)**. Finally, debris was removed and viable HSPCs defined as CD34^+^CD45^dim^SSC^low^ on a FSC vs. SSC plot **(E)**.

A SPFC method validated by the International Society of Hematotherapy and Graft Engineering (ISHAGE) was used to identify HSPCs (defined as CD34^+^CD45^dim^SSC^low^) in whole blood. Whole blood (100 µL) was stained with anti-human CD34-PE (clone 581), anti-human CD45-FITC (clone 2D1), CD38-BV421 (clone HB-7), and a viability exclusion dye (7-amino-actinomycin D, 7-AAD) at room temperature, in the dark, for 30 min and then 2 mL of red blood cell lysis buffer added for 10 min. All samples were analysed within 1 h. HSPCs were enumerated using a Boolean gating strategy based on three key criteria: positive expression of CD34, moderate expression of CD45 and low side scatter (Figure 2). A minimum of 100 HSPC events were acquired in the final gate in accordance with ISHAGE guidelines and data expressed as the concentration of HSPCs in whole blood (cells/µL), fold change or total area under the curve (AUC), calculating using the trapezoid method (Gagnon and Peterson, 1998). The frequency of HSPCs expressing CD38 was also calculated.

#### 2.5.2 Double platform flow cytometry

Cryopreserved PBMCs were thawed by submerging half of the vials containing approximately ≈20 million cells for 1 min and then gently pouring the cells into sterile 15-mL falcon tubes. Cells were washed 3 times with RPMI supplemented with 20% FBS, 100 U/mL Pen-Strep, and 2 mM Glutamine. PBMCs (2 × 10^6^) were stained with anti-human CD45-FITC (clone 2D1), anti-human CD34-PE (clone 581), anti-human CXCR4-APC (clone 12G5), anti-human VLA4-AF (clone Hu114), anti-human CD3-FITC (clone UCHT1), anti-human CD56-PE (clone 5.1H11) and anti-human CD16-APC (clone 3G8) and 7-AAD on ice, in the dark for 30 min. These cells were then washed three times in FACS buffer (500 mL of D-PBS, 2 mM EDTA, 0.1% Sodium Azide, and 1 mM FBS) for 5 min at 500 × *g* and 4°C before data acquisition.

A DPFC approach was used to calculate the concentration of HSPCs, CD3^+^ T cells, CD56^dim^ and CD56^bright^ NK cells in peripheral blood by coupling their frequency in the PBMC fraction determined by flow cytometry to a whole blood lymphocyte count from the same sample using an automated haematology analyser (*Yumizen H500*, *Horiba*, *Kyoto*, *Japan*). Lymphocytes were identified by forward scatter (FSC) vs. side scatter (SSC), and then CD3^+^ events used to enumerate T cells. In the CD3^-^ gate, a bivariate plot between CD16 and CD56 was used to define cytolytic (CD16^+^ CD56^dim^) and regulatory (CD16^-^ CD56^bright^) natural killer (NK) cells. A minimum of 1 × 10^6^ lymphocyte events were acquired, resulting in approximately 1 × 10^3^, 1 × 10^6^, 5 × 10^4^, and 1 × 10^3^ events in the HSPC, CD3^+^, CD56^dim^ and CD56^bright^ gates, respectively. Data were expressed as concentration in peripheral blood (cells/µL) or fold change. Within the HSPC gate, the frequency (%) of CXCR-4^+^ and VLA-4^+^ HSPCs were calculated and used to determine a peripheral blood concentration (cells/µL) for each cell type. The cell surface expression of CXCR-4 and VLA-4 on gated HSPCs was then determined using the Geometric Mean Fluorescence Intensity (GeoMean).

### 2.6 Correction of cell concentrations for changes in blood volume

Changes in cell concentrations vs. rest measured using DPFC (T cells, NK cell subsets, HSPCs, CXCR-4^+^ HSPCs and VLA-4^+^ HSPCs), automated haematology analysis (total white blood cells, neutrophils, lymphocytes, monocyte), and SPFC (HSPCs) were adjusted for changes in blood volume using the formula proposed by Matomäki et al., 2000 (Matomäki et al., 2018).

### 2.7 Enzyme-linked immunosorbent assays

The concentration of soluble adhesion molecules CXCL-12/SDF-1 (stromal cell-derived factor-1) and VCAM-1 (Vascular cell adhesion molecule-1) were determined in plasma using enzyme-linked immunosorbent assay (ELISA) kits purchased from Bio-techne (*Minneapolis*, *United States*, *assay sensitivity*, *CXCL-12: 18* *pg/mL and VCAM-1: 0.6* *ng/mL*). All samples were analysed in duplicate and concentrations were obtained from a standard curve of known CXCL-12 and VCAM-1 concentrations and adjusted for changes in plasma volume (Dill and Costill, 1974). The manufacturer reported intra- (*CXCL-12: 3.6% and VCAM-1:* 0.6%) and inter-assay (*CXCL-12: 10.3% and VCAM-1:* 7.0%) variability that aligned with our laboratory.

### 2.8 Statistical analysis

Statistical analyses were performed using GraphPad Prism 10.0.3 analysis software (*San Diego*, *CA*). Data at all timepoints were checked for normal distribution using the Shapiro-Wilk test. Normally distributed variables were analysed over time (Rest, End of intervals 1–4, 5-minute and 10-minute post exercise) and across Trials (MICE, HV-HIIE, and LV-HIIE) by mixed-effects two-way analysis of variance (Two-way ANOVA). Data that weren’t normally distributed were analysed using Wilcoxon or Kruskal-Wallis’s test. Post hoc analyses of any interaction effects (Time × Trial) were performed by a test of multiple comparisons, with either Tukey or Dunn’s test, depending on variable normality. All values are presented as means ± standard deviation (SD). Statistical significance was accepted at the *P* < 0.05 level. Effect sizes were calculated where appropriate by dividing the difference between the means by the pooled standard deviations. An effect size of 0.2 was considered the minimal value for a meaningful difference, 0.5 for moderate and 0.8 for large (Cohen, 1988). The relationship between HSPC concentrations determined by SPFC and DPFC methods was evaluated by calculating a Pearson correlation coefficient and agreement of these methods by formulating a Bland-Altman plot. For the latter, mean concentrations of both methods (*x*-axis) were plotted against the difference between these values (SPFC–DPFC, *y*-axis). This enabled identification of systemic differences between these two quantitative methods, including degree of bias and outliers. The limits of agreement [“lower limit” = mean difference – (1.96 × standard deviation of difference) and “upper limit” = mean difference + (1.96 × standard deviation of difference)] were calculated according to 95% confidence intervals.

## 3 Results

### 3.1 Physiological responses and subjective perceptions during experimental trials

There were no significant differences in resting HR [F (2, 30) = 0.09, *P* = 0.91] and participants weight remained stable across all three experimental trials [F (2, 30) = 0.01, *P* < 0.99]. There was no difference between anxiety and sleep quality between the three experimental trials (Supplementary Table S2). These variables were therefore not used as covariates in subsequent ANOVA analyses.

By design, average power output [F (2, 30) = 46.54, *P* < 0.0001], peak HR (HR_peak_) [F (2, 30) = 115.50, *P* < 0.0001], HR_max_ [F (2, 30) = 354.00, *P* < 0.0001] and average RPE [F (2, 30) = 25.08, *P* < 0.0001] were greater throughout LV-HIIE > HV-HIIE > MICE (Table 1) and estimated total energy expenditure was greater in MICE > HV-HIIE > LV-HIIE [F (2, 30) = 25.48, *P* < 0.0001]. A repeated measures ANOVA indicated significant differences in average HR after each interval (Time × Trial Interaction: F (6, 90) = 2.60, *P* = 0.02). *Post-hoc* analysis revealed that heart rate peaked after the second interval during HV-HIIE (155.60 ± 7.78, *P* < 0.0001), the third interval during LV-HIIE (171.90 ± 6.43, *P* < 0.0001) and remained stable above rest during MICE (125.18 ± 5.33, *P* > 0.99). Within each trial, power output and RPE remained consistent over the 30-minute trial or between intervals (Time × Trial Interaction: *P* > 0.05). There was no significant difference in the affective response between the three trials measured by the feeling scale [F (2, 30) = 1.21, *P* = 0.31] (Table 1).

**TABLE 1:** Physiological responses during each experimental trial.

Cycling trial				
Parameter	MICE	HV-HIIE	LV-HIIE	*P*-value
Average power output (W)	132 ± 20^a^ ^,^ ^b^	215 ± 32^a^ ^,^ ^c^	271 ± 45^b^ ^,^ ^c^	<0.0001
Relative maximal power (%)	42 ± 5^a^ ^,^ ^b^	69 ± 3^a^ ^,^ ^c^	87 ± 3^b^ ^,^ ^c^	<0.0001
HR_peak_ (bpm)	131 ± 7^a^ ^,^ ^b^	162 ± 7^a^ ^,^ ^c^	175 ± 7^b^ ^,^ ^c^	<0.0001
HR_max_ (%)	70 ± 2^a^ ^,^ ^b^	84 ± 3^a^ ^,^ ^c^	95 ± 2^b^ ^,^ ^c^	<0.0001
Estimated energy expenditure (kcal)	221 ± 43^a^ ^,^ ^b^	152 ± 46^a^ ^,^ ^c^	87 ± 45^b^ ^,^ ^c^	<0.01
Average RPE	11.5 ± 0.6^a^ ^,^ ^b^	14.6 ± 0.9^a^ ^,^ ^c^	16.7 ± 1.0^b^ ^,^ ^c^	<0.0001
Average feeling scale	3.0 ± 1.5	2.4 ± 1.7	1.7 ± 2.4	>0.05

Data displayed as mean ± SD.

Abbreviations: MICE, moderate intensity continuous exercise; HV-HIIE, high volume-high intensity interval exercise; LV-HIIE, low volume-high intensity interval exercise; HR_peak_, peak heart rate; RPE, rating of perceived exertion.

^a^ Significant difference between MICE and HV-HIIE (*P* < 0.05).

^b^ Significant difference between MICE and LV-HIIE (*P* < 0.05).

^c^ Significant difference between HV-HIIE and LV-HIIE (*P* < 0.05).

### 3.2 SPFC to determine peripheral blood HSPC concentrations

**FIGURE 3:**
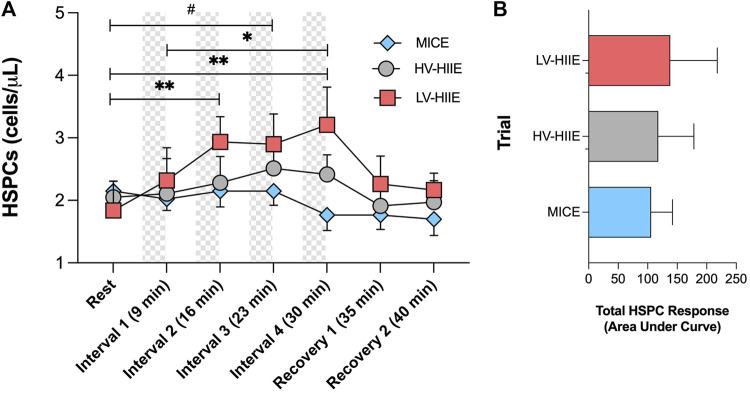
Changes in peripheral blood HSPC concentrations **(A)** and total area under the curve HSPC response **(B)** across seven timepoints of MICE (blue bars), HV-HIIE (grey bars) and LV-HIIE (red bars) enumerated by SPFC. Values are means ± SD. * and ^#^ indicate significant differences between timepoints in LV-HIIE and HV-HIIE respectively: *P < 0.05, **P < 0.01, ^#^ *P* < 0.05.

Changes in peripheral blood HSPC concentrations in response to trials determined by SPFC are shown in Figure 3. A Time × Trial interaction effect was observed [F (12, 180) = 2.31, *P* = 0.01], indicating no change in HSPC concentration during MICE and a significant increase after two intervals of LV-HIIE (Rest: 1.84 ± 1.55 vs. Interval 2: 2.94 ± 1.34, *P* = 0.005), and three intervals of HV-HIIE (Rest: 2.05 ± 0.86 vs. Interval 3: 2.51 ± 1.05, *P* = 0.04), although this was only sustained through to the end of Interval 4 during LV-HIIE. A cumulative increase was observed during LV-HIIE only, with HSPC concentration significantly greater following Interval 4 (3.21 ± 2.00) than Interval 1 (2.32 ± 1.73, *P* = 0.03). A *post hoc* analysis comparing peak HSPC concentrations at Interval 4 (30 min) demonstrated a significant difference between LV-HIIE vs. MICE (LV-HIIE: 3.21 ± 2 vs. MICE: 1.76 ± 0.82, *P* = 0.02). In all trials, HSPC concentrations decreased to resting levels within 5 min of cycling cessation. Analysis of changes in HSPC concentrations over time by calculating AUC revealed no statistically significant differences between trials (LV-HIIE: 138 ± 79.71, HV-HIIE: 117.90 ± 60.46, MICE: 105.90 ± 36.23) (Figure 3B). The Cohen’s d effect size for MICE vs. LV-HIIE was 0.3, whereas comparisons between other trials were <0.2. The majority of circulating HSPCs were CD38^+^ (95.23% ± 10.91) and the frequency of both CD38^+^ and CD38^-^ HSPCs did not change throughout MICE, HV-HIIE or LV-HIIE (*P* > 0.05, see Supplementary Figures S1C, D). The concentration of CD38^+^ HSPCs increased after LV-HIIE only (Rest: 1.66 ± 1.51 vs. Interval 4: 2.82 ± 2.04, *P* < 0.0001), with no changes in CD38^-^ HSPCs observed (Supplementary Figures S1A, B).

### 3.3 DPFC to determine peripheral blood HSPC concentrations

Changes in peripheral blood HSPC concentrations in response to trials determined by DPFC are shown in Figure 7B. A repeated measures ANOVA revealed a significant Time × Trial interaction effect [F (2, 30) = 10.27, *P* = 0.0004]. *Post-hoc* analyses illustrated an increase in HSPC concentration after Interval 4 of LV-HIIE relative to rest (Rest: 2.45 ± 1.09 vs. Interval 4: 3.72 ± 1.20, *P* < 0.0001) and HV-HIIE (Rest: 2.20 ± 0.88 vs. Interval 4: 3.26 ± 1.28, *P* < 0.0001), but not after MICE (Rest: 2.43 ± 0.85 vs. Interval 4: 2.57 ± 1.10, *P* > 0.99). There were significant differences in post-exercise HSPC concentrations between LV-HIIE and MICE (*P* = 0.04), but not HV-HIIE vs. MICE (*P* = 0.41) or LV-HIIE vs. HV-HIIE (*P* = 0.97).

### 3.4 Immune cell subsets

**FIGURE 4:**
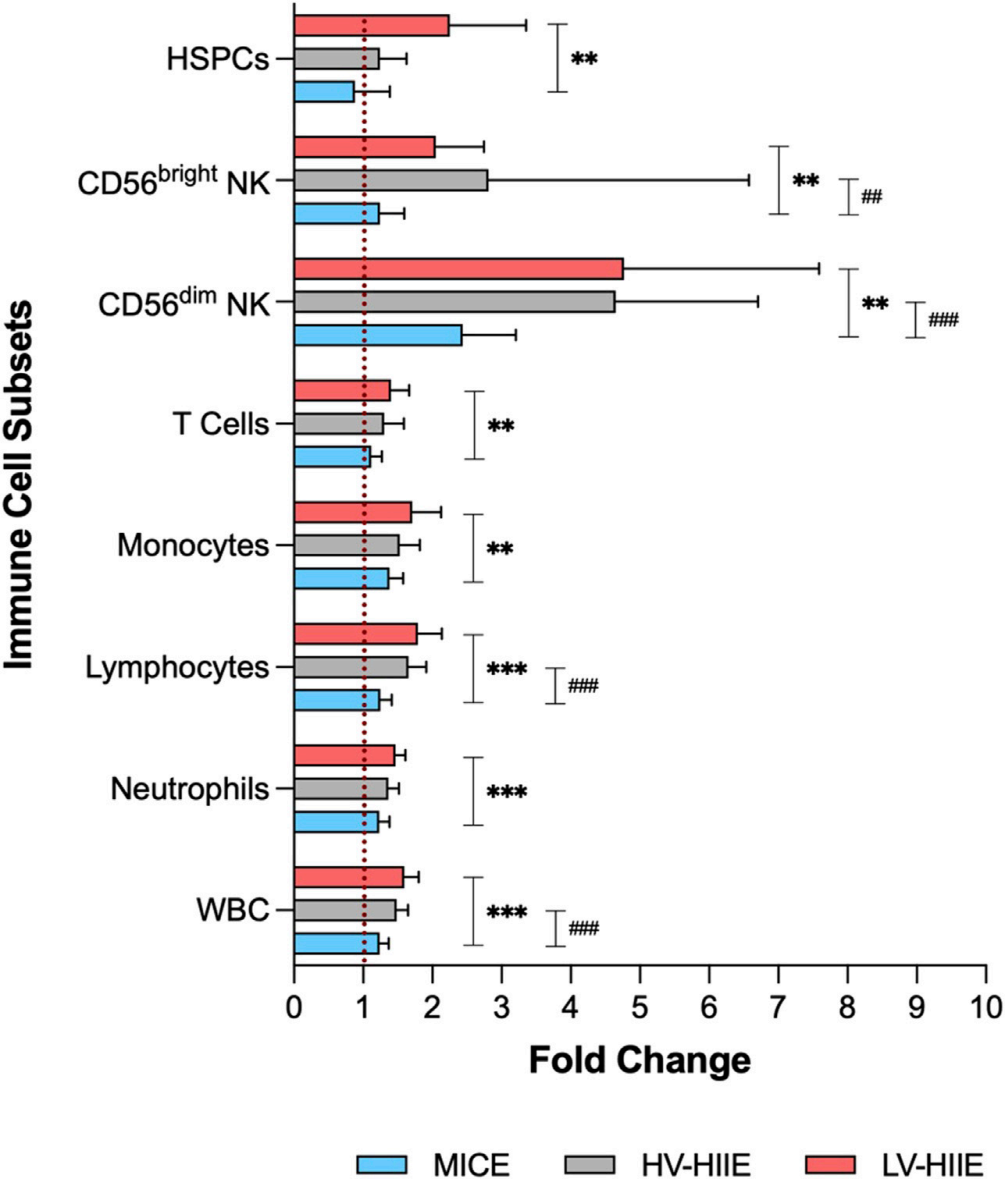
Fold change in immune cell subset concentrations (WBC, neutrophils, lymphocytes, monocytes, T cells, CD56^dim^ NK cells, CD56^bright^ NK cells and HSPCs) after Interval 4 (30 min) relative to rest in MICE (blue bars), HV-HIIE (grey bars) and LV-HIIE (red bars). * and # indicate significant differences between LV-HIIE vs. MICE and HV-HIIE vs. MICE respectively: **P < 0.01, ***P < 0.0001, ^##^ *P* < 0.01, ^###^ *P* < 0.0001.

Data obtained from automated haematology analysis and further phenotyping using flow cytometry indicated Time × Trial Interactions for peripheral blood concentrations of all immune cell subsets (full details and statistical output in Supplementary Table S1). A comparison between Rest and Interval 4 (30 min) only indicated that the concentration of white blood cells was not significantly different across trials, but analysis of daughter populations revealed that monocyte, lymphocyte, and CD56^dim^ NK cell concentrations were greater immediately after LV-HIIE compared to MICE. The concentration of CD56^dim^ NK cells was greater after HV-HIIE > MICE, and for monocytes, was greater after LV-HIIE > HV-HIIE. Following adjustment of all cell concentrations for changes in blood volume, all Time effects remained significant, but there were no Time × Trial interaction effects noted for blood volume adjusted neutrophil, monocyte, and T cell concentrations. For visualisation of these changes across all immune cell subsets and trials, fold changes between Rest and Interval 4 (30 min) are depicted in Figure 4, and a comparison of the magnitude of change between cell subsets detailed in Supplementary Figures S2A–C. Fold change of HSPCs was not greater than any other subset and mirrored the general leukocyte pattern across all trials. In contrast, fold change of CD56dim NK cells was mostly greater than every subset across all trials (all *P* < 0.01), whereas fold change of CD56^bright^ NK cells was significantly greater than neutrophils and T cells after LV-HIIE and HV-HIIE only (all *P* < 0.01). Finally, fold change of lymphocytes was greater than neutrophils after LV-HIIE and HV-HIIE only (all *P* < 0.01), and greater than monocytes after HV-HIIE only (*P* < 0.01).

### 3.5 Bone marrow homing potential of HSPCs

**FIGURE 5:**
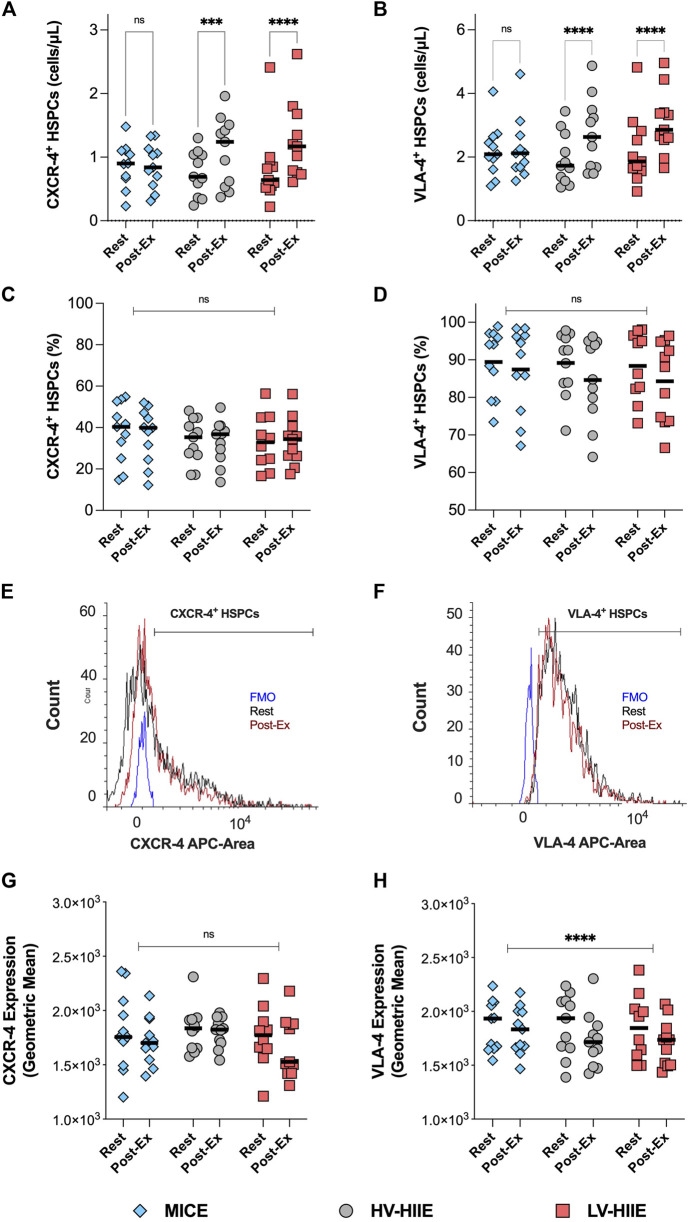
Changes in the peripheral blood concentration **(A,B)**, frequency **(C,D)** and cell surface expression **(G,H)** of CXCR-4 and VLA-4 on gated HSPCs between rest and Interval 4 (30 min) in MICE (blue bars), HV-HIIE (grey bars) and LV-HIIE (red bars). All data was obtained using DPFC and automated hematology analysis. The cell surface expression of CXCR-4 and VLA-4 on HSPCs at rest (black histogram) and Interval 4 (30 min) or ‘Post-Ex’ (red histogram) were established by determining the positive population with a fluorescence minus one (FMO) control (blue histogram) and then calculating the Geometric mean **(E,F)**. Values are means ± SD. * indicates significant differences between Pre- and Post-Ex, representing pairwise comparisons in each trial **(A,B)** and in all trials **(C,D,G,H)**: ***P < 0.001, ****P < 0.0001. Ns indicates no significant differences between timepoints or trials: *P* > 0.05. The “Interval 4 (30 min)” timepoint is represented as “Post-Ex.”

Changes in the peripheral blood concentration (cells/µL, Figures 5A, B) and frequency (Figures 5C, D) of CXCR-4^+^ and VLA-4^+^ HSPCs after each trial are reported in Figure 5. The cell surface expression of CXCR-4 and VLA-4 on gated HSPCs are depicted by a representative histogram (Figures 5E, F) and the average GeoMean data reported (Figures 5G, H).

A repeated measures ANOVA revealed a significant Time × Trial interaction for CXCR-4^+^ [F (2, 30) = 8.17, *P* = 0.002] and VLA-4^+^ HSPC concentrations [F (2, 30) = 7.46, *P* = 0.002]. *Post-hoc* analyses revealed that compared with rest, CXCR-4^+^ and VLA-4^+^ HSPC concentrations increased after LV-HIIE (both *P* < 0.0001) and HV-HIIE (both < *P* = 0.001), but not MICE (both *P* > 0.05). Within the HSPC population, there were no differences in the frequency of CXCR-4^+^ or VLA-4^+^ HSPCs in response to the trials (*P* < 0.05). When comparing changes in the cell surface expression levels of these receptors, there was a significant decrease in VLA-4 (Time Effect: *P* < 0.0001), but no change in CXCR-4 after all trials (Time Effect: *P* = 0.66).

### 3.6 Chemokine CXCL-12 and VCAM-1 concentrations

**FIGURE 6:**
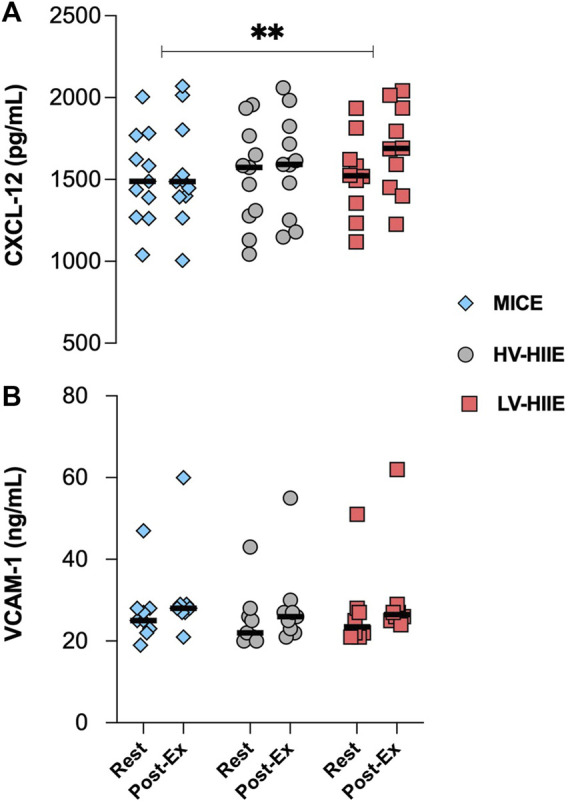
Changes in the plasma concentration of CXCL-12 **(A)** and VCAM-1 **(B)** between Rest and Interval 4 (30 min) of MICE (blue bars), HV-HIIE (grey bars) and LV-HIIE (red bars). Values are means ± SD. * indicates a significant effect of Time: **p < 0.01. The “Interval 4 (30 min)” timepoint is represented as “Post-Ex.”

Changes in plasma volume adjusted chemokine concentrations in response to each trial are presented in Figure 6. A repeated measures ANOVA revealed a significant effect of Time for CXCL-12 concentration [F (1, 29) = 10.18, *P* = 0.003], but no interaction effect (*P* = 0.11). There were no changes in plasma VCAM-1 (*P* = 0.80) concentration in response to all cycling trials. There were no associations between changes in plasma chemokine and HSPC concentrations across any of the trials (Supplementary Figures S3A, B).

### 3.7 Comparison of flow cytometric methods to determine HSPC concentrations

**FIGURE 7:**
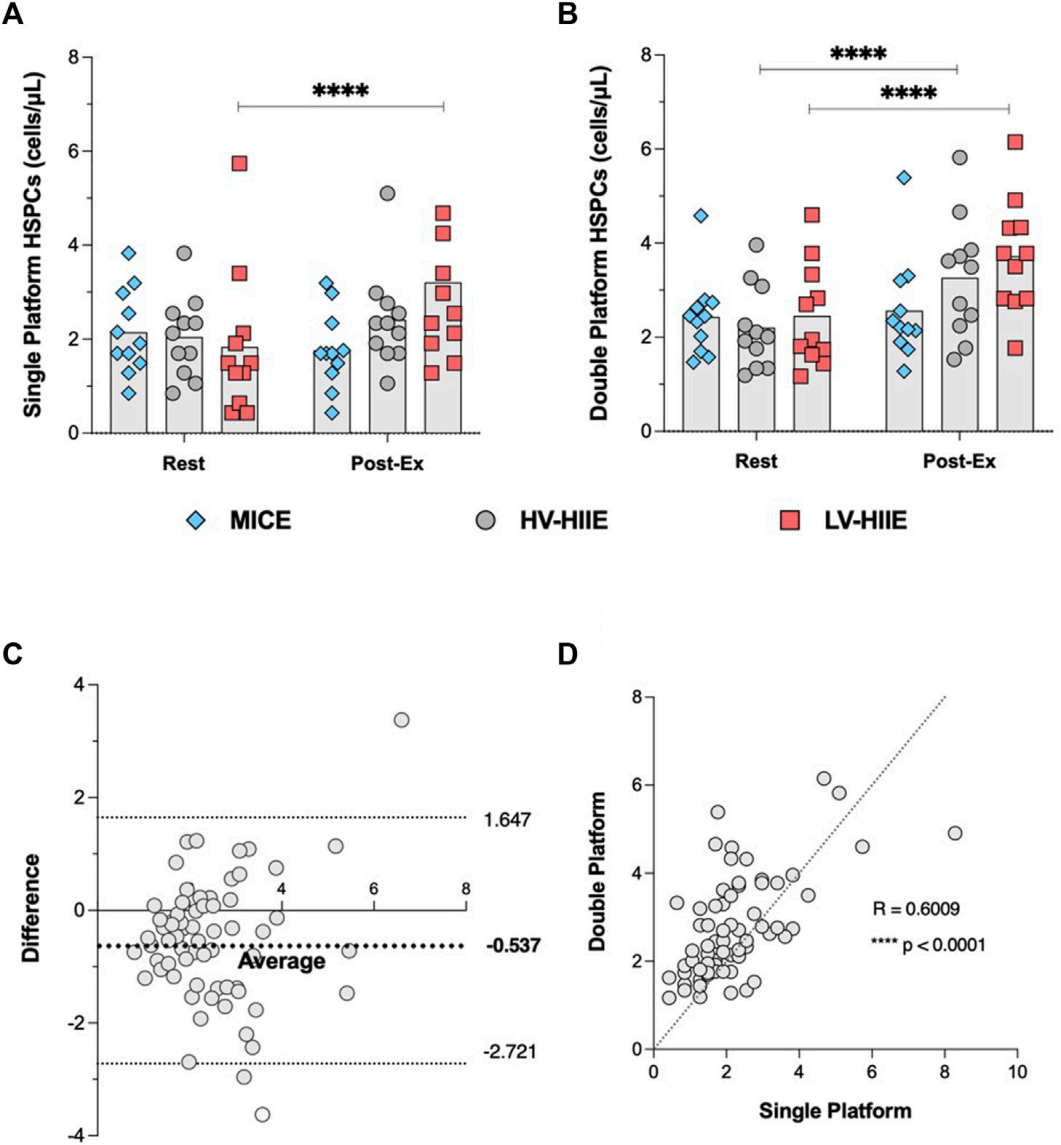
Changes in peripheral blood HSPC concentrations determined by SPFC **(A)** and DPFC **(B)** between Rest and Interval 4 (30 min) in MICE (blue bars), HV-HIIE (grey bars) and LV-HIIE (red bars). The agreement between SPFC and DPFC was established by determining a Pearson correlation coefficient **(C)** and Bland-Altman plot **(D)**. Values are means ± SD. * indicates significant differences between Pre- and Post-Ex: ****p < 0.0001. The “Interval 4 (30 min)” timepoint is represented as “Post-Ex.”

A Pearson’s correlation indicated that there was a significantly positive relationship (Figure 7C) between SPFC and DPFC methods (R = 0.60, *P* < 0.0001). Furthermore, Bland-Altman analysis revealed no systemic differences between SPFC and DPFC methods. Figure 7D indicated that most differences (63 of 66 measurements) between SPFC and DPFC fell within the “lower” (−2.721) and “upper” (1.647) limits of agreement. The mean difference was −0.537 cells/µL between data pairs. A linear regression analysis between the mean of the data pairs vs. mean difference indicated no significant difference between methods [F (1, 64) = 1.28, *P* = 0.26]. Moreover, main effects and *post hoc* analyses comparing HSPC concentration between Rest and Interval 4 across all three experimental trials revealed similar results from both methods.

## 4 Discussion

The results of the current study indicate that brief cycling intervals of high intensity (LV-HIIE and HV-HIIE), but not moderate intensity continuous cycling (MICE) mobilised HSPCs into peripheral blood of healthy young males. HSPC concentrations determined by SPFC were elevated above rest after just two intervals of LV-HIIE (4 min) and three intervals of HV-HIIE (12 min). However, immediately following completion of Interval 4, HSPC concentration was only elevated above rest during LV-HIIE, returning to resting concentration within 5 min. These responses were exclusive to CD38^+^ HSPCs, with no changes in the CD38^-^ fraction observed. The frequency of HSPCs expressing bone marrow homing receptors CXCR-4 and VLA-4 were unaltered, but the cell surface expression of VLA-4 decreased after all cycling trials, indicating marginally altered bone marrow homing propensity of exercise-mobilised HSPCs. Accompanying these changes, both HIIE trials evoked more marked increases in the peripheral blood concentration of cytolytic CD56^dim^ NK cells compared to MICE. Taken together, these data indicate that just 4–12 min of high intensity cycling intervals can enrich peripheral blood with more HSPCs and CD56^dim^ NK cells compared to 30 min of moderate intensity continuous cycling. Given the importance of these cells in context of HSPC transplantation, and the low volume of cycling needed to evoke these changes, these findings provide a rationale to investigate the impact of interval cycling after prior G-CSF treatment to examine HSPC collection efficacy.

Previous studies have reported increases in peripheral blood HSPC concentrations after bouts of steady state exercise (Schmid et al., 2021b) and HIIE (Krüger et al., 2015; O’Carroll et al., 2019). Data supports “intensity” to be a more prominent driver of HSPC mobilisation than “duration” of a steady state exercise bout (Agha et al., 2018); however, the relationship between these variables when comparing bouts of HIIE is unclear. Whereas “all-out” HIIE over a cumulative total of 120 s has been reported to increase HSPC concentration (O’Carroll et al., 2019), 15 min of intervals at a lower intensity (5 × 3 min at 90% of peak power) were needed to evoke a similar change (Krüger et al., 2015), with 10 min not sufficient (10 × 1 min at 90% of maximal heart rate) (Nederveen et al., 2020). Differences in participant demographics, timing between intervals, and criteria used to define exercise “intensity” may partly explain some of the discrepancies noted between these studies. Moreover, studies to date have not directly compared how different volumes of HIIE impact HSPC mobilisation, which was a primary purpose of the current study. Given the potential translation of HIIE into a PBSC setting, the present study compared cycling protocols that aligned with criteria used to define clinical HIIE (Weston et al., 2014; Taylor et al., 2019). We adopted the lower (85%) and upper intensity (95%) limits of these criteria for maximal heart rate to design HV-HIIE and LV-HIIE protocols respectively. A further novel element of the study was employing serial blood sampling to evaluate changes after each interval of HIIE and time-matched samples during MICE. This approach enabled us to determine that just 4 min of LV-HIIE and 12 min of HV-HIIE significantly increased HSPC concentrations compared to rest. Comparatively, no changes were observed across seven timepoints over 30 min of moderate intensity continuous exercise (MICE), although total AUC between trials was not statistically different. Despite no statistically significant differences between LV-HIIE and HV-HIIE, HSPC concentrations were only greater after Interval 4 (30 min) of LV-HIIE vs. MICE and not HV-HIIE vs. MICE. Moreover, the lower volume of total work performed in LV-HIIE compared to HV-HIIE (Table 1) and more rapid initial increase in HSPCs indicate that the “intensity” of HIIE is a more prominent determinant of HSPC mobilisation than “duration.” The approximate 2-fold increase in HSPC concentration observed after LV-HIIE aligns with concentrations reported from previous studies evaluating intervals and steady state exercise of much longer duration (Baker et al., 2017; Niemiro et al., 2017; Agha et al., 2018). Although maximal intensity cycling intervals can evoke HSPC mobilisation to this degree (O’Carroll et al., 2019), our data indicate that low volume cycling at a more manageable intensity elicits a comparable increase.

In the present study, higher average power output, subjective exertion and HR_peak_ (Table 1) in LV- HIIE > HV-HIIE > MICE reflected greater physiological exertion that would likely elicit greater increases in systemic adrenaline concentration (Simpson et al., 2021) and therefore HSPC mobilisation, corroborating previous work (Agha et al., 2018). The observed intensity-dependent changes in HSPCs were also observed across other immune cells subsets (Supplemental Table S1). Notably, the increased concentrations of lymphocytes and CD56^dim^ NK cells were greater in response to LV-HIIE vs. MICE. The increased concentration of monocytes was greatest in LV-HIIE vs. HV-HIIE and MICE; however, this was driven by greater reductions in blood volume during LV-HIIE rather than greater cell mobilisation. It is well established that single bouts of exercise elicit non-uniform mobilisation of immune cells into peripheral blood, which is intensity dependent (Baker et al., 2017). Cells with high effector functions (e.g., non-classical monocytes, effector memory T cells and CD56^dim^ NK cells are preferentially mobilised relative to naïve subsets under the actions of β2 adrenergic (Graff et al., 2018) and cytokine signalling (Bay et al., 2020). Although extensive phenotyping was not carried out in the present study, CD56^dim^ NK cells had markedly higher ingress than any other cell subset (Figure 4), illustrating preferential mobilisation of potent cytolytic cells, particularly during HIIE.

### 4.1 Perspectives and clinical considerations

The concept of implementing exercise in a PBSC setting has been proposed previously (Emmons et al., 2016b; Simpson et al., 2017), and yet practical steps to facilitate this have not been taken. This first begins with providing empirical data to support an appropriate type of exercise for a broad range of autologous and allogeneic donors, before evaluating its clinical utility. The present study reports that HIIE can enrich peripheral blood with higher numbers of multiple immune cell subsets to a greater degree than MICE, most notably HSPCs and CD56^dim^ NK cells. However, in line with previous studies (Schmid et al., 2021b), the increase in HSPC concentration observed was transient, with resting concentration restored within 5 min. PBSC collections take approximately 3–4-hour and the peak HSPC concentration of 3.2 ± 2.0 cells/µL during LV-HIIE (Figure 3A) reported herein falls short of the minimum collection threshold of 10 cells/µL following G-CSF treatment (Stroncek et al., 1997). Furthermore, when expressing HSPC data relative to body mass, despite a significant increase after LV-HIIE in the absence of G-CSF, HSPC dose after the trial was approximately 10-fold short of the 2 × 10^6^ HSPCs/kg collection threshold required for successful engraftment after autologous transplantation (*data not shown*) (Baker et al., 2017). Therefore, bouts of exercise in isolation are insufficient to evoke clinically meaningful changes in HSPCs. Furthermore, CD38^+^, but not CD38^-^ HSPC concentrations increased after LV-HIIE in the present study (Supplementary Figures S1A, B). Although engraftment success is predicted clinically by CD34^+^ count alone (Duggan et al., 2000; Allan et al., 2002; Basquiera et al., 2006), a higher number and frequency of pluripotent CD38^-^ HSCs are mobilised from the bone marrow following G-CSF therapy (Bonig and Papayannopoulou, 2012) and predict more favourable engraftment success vs. CD38^+^ HSPCs (Astori et al., 2001). It is noteworthy that the majority of circulating HSPCs in the present study were progenitor cells (≈95% at rest and after exercise, Supplementary Figures S1C, D). Enrichment of peripheral blood with CD38^-^ HSPCs after G-CSF treatment results in a proportion of HSPCs residing in marginal pools of the circulation, thus not available for collection (Simpson et al., 2017). Presently, it is unclear whether exercise could dislodge marginalised CD38^+^ or CD38^-^ HSPCs into the blood compartment during apheresis.

Beyond the importance of the harvested cell number, the engraftment potential of HSPCs and other collected cells are important. In the present study, engraftment potential of HSPCs was evaluated by quantifying cell surface expression of CXCR-4 and VLA-4. Although expression is known to be variable across individuals, our data aligned with mean CXCR-4^+^ (≈40%) (Taylor et al., 2021) and VLA-4^+^ (≈68.5%) (Bellucci et al., 1999) HSPC frequencies in the absence of prior G-CSF treatment reported in other studies. The concentration, but not frequency of HSPCs expressing bone marrow homing receptors CXCR-4 and VLA-4 were increased after HIIE, but not MICE (Figures 5A–D). Previous studies support these findings, reporting an increase in the concentration, but not frequency of peripheral blood CXCR-4^+^ HSPCs (Taylor et al., 2021), and VLA-4^+^ T, B, NK cells (Miles et al., 1998) after steady state incline walking and resistance exercise respectively. Therefore, CXCR-4^+^ and VLA-4^+^ HSPC mobilisation during HIIE likely reflects non-specific demargination of these cells from marginal pools along with all leukocytes (Adams et al., 2011). Additionally, we observed a decrease in the cell surface expression of VLA-4, but not CXCR-4 after all cycling trials (Figures 5G–H). The cell surface expression of CXCR-4 has been reported to be augmented on circulating T (Okutsu et al., 2005) and NK cells (Okutsu et al., 2014) after steady state exercise and associated with elevated tissue homing and subsequent lymphocytopenia. Expression changes of CXCR-4 and VLA-4 after bouts of exercise are evidently specific to each cell type and dependent on their microenvironment, notably chemokine cues. We observed no relationship between the expression of these receptors with systemic changes in CXCL-12 and VCAM-1 concentrations, which displayed the reverse pattern (Figures 6A, B) (Niemiro et al., 2017), and thus local tissue chemokine cues might explain the decrease in VLA-4 expression on HSPCs. The latter implies that the bone marrow homing propensity of HSPCs was impaired after exercise relative to circulating HSPCs at rest; however, its impact on engraftment potential is presently unclear. Engraftment potential is also impacted by immune composition, notably CD56^dim^ NK cells, which were ≈4-fold higher after HIIE vs. MICE (Figure 4). Higher numbers of CD56^dim^ NK cells have been reported to lower the risk of several clinical endpoints (e.g., post-transplant viral infections and GvHD) following allogenic HSPC transplantation by enhancing the recipient’s adaptive immune responses (Porrata et al., 2004).

Collectively, repeated intervals of LV-HIIE incrementally increased the concentration of peripheral blood HSPCs, albeit with a modulated bone marrow phenotype, to a greater degree than moderate intensity steady state cycling. These changes were accompanied by an increase in clinically relevant CD56^dim^ NK cells in peripheral blood at the end of interval 4.

### 4.2 Comparison of SPFC and DPFC methods

A secondary aim of the present study was to evaluate the relationship and systemic bias between SPFC and DPFC methods for enumerating HSPCs in peripheral blood before and after bouts of cycling. DPFC is more commonplace in studies evaluating changes in HSPC concentration after exercise, but high variability has been reported in some multi-site studies (Gratama et al., 2003) whereas SPFC is the clinically accepted method used during PBSC collections, established by ISHAGE (Sutherland et al., 1996). The same general pattern of response was observed between methods (Figures 7A, B), with the exception that post-exercise HSPC concentrations were significantly greater than rest after LV-HIIE and HV-HIIE vs. MICE using DPFC (Figure 7B), whereas this trend was only significant for LV-HIIE using SPFC (Figure 7A). A significant moderate correlation was observed between methods (Figure 7C), and there was no significant systemic bias between methods (Figure 7D). Whereas both quantitative methods appear acceptable depending on logistics of a research study design, a mean difference of −0.537 cells/µL might be considered clinically meaningful given that the collection threshold for initiating apheresis is >10 cells/µL (Panch et al., 2017). Regardless of method, these data collectively indicate that HIIE evoked greater mobilisation of HSPCs than MICE.

### 4.3 Strengths, limitations and future perspectives

Using a randomised crossover design and serial blood sampling, this study employed internationally validated guidelines to enumerate peripheral blood HSPC concentrations after bouts of interval vs. continuous cycling; however, this study was not without limitations. A low recruitment rate following easing of COVID-19 restrictions resulted in our study population including only male participants, thus not biologically, socially or clinically representative. From a physiological perspective, our data indicate that just 4 min of LV-HIIE elicited an increase in peripheral blood HSPCs greater than 30 min steady state cycling, but at present, the translation of these findings are limited. It is evident that clinical trials are required to evaluate whether cycling during apheresis (with prior G-CSF) can improve HSPC collection efficacy. These trials should be preceded by studies first examining whether cycling throughout a PBSC collection procedure (≈3 h) sustains HSPC concentrations and the effect on immune composition. Beyond the physiological potential of exercise to maximise the number of these cells, future work is required to evaluate the feasibility and acceptability of such an approach for allogeneic and autologous donors undergoing G-CSF treatment. Cycling protocols such as LV-HIIE and HV-HIIE could be unfavourable for allogenic donors in the general population who do not exercise regularly and not recommended for autologous donors suffering with immunosuppressive disorders (Baumann et al., 2011). Despite the physiological strain during LV-HIIE being markedly greater than MICE, the enjoyment level was equal across all exercise trials (Table 1). This provides some basic subjective data to support the use of these protocols in healthy young males, but implementation of these intervals over 3 h in different population groups, albeit with longer rest periods, warrants investigation.

## 5 Conclusion

In conclusion, the present study revealed that 2 × 2-minute bouts of LV-HIIE and 3 × 4-minute bouts of HV-HIIE were sufficient to increase peripheral blood CD38^+^ HSPC concentrations above rest, but not in response to 30 min of continuous cycling at moderate intensity. At the end of the trials, HSPC concentration was only elevated above rest in the LV-HIIE trial. Furthermore, peripheral blood was enriched with higher numbers of CD56^dim^ NK cells after bouts of HIIE vs. MICE. Collectively, these data indicate that exercise intensity was a more prominent factor than duration in driving the mobilisation of immune cells with relevance to HSPC transplantation. With only 4 min of high intensity cycling intervals eliciting these changes, future studies should evaluate the clinical effectiveness of HIIE within a PBSC donation setting.

## Figures and Tables

**Table:** 

**PBSC**	Peripheral blood stem cell
**HSPC**	Haematopoietic stem and progenitor cell
**HIIE**	High intensity interval exercise
**MICE**	Moderate intensity continuous exercise
**HV-HIIE**	High volume-high intensity interval exercise
**LV-HIIE**	Low volume-high intensity interval exercise
**HR**_**max**_	Maximum heart rate
**CXCR-4**	C-X-C chemokine receptor 4
**VLA-4**	Very Late antigen 4
**NK cell**	Natural killer cell
**G-CSF**	Granulocyte colony stimulating factor
**HLA**	Human leukocyte antigen
**GvHD**	Graft-versus-host disease
**CXCL-12**	C-X-C motif chemokine 12
**VCAM-1**	Vascular cellular adhesion molecule 1
**β2**	Beta 2
**ISHAGE**	The International Society of Hematotherapy and Graft Engineering
**SPFC**	Single platform flow cytometry
**DPFC**	Double platform flow cytometry
**GPPAQ**	General practice physical activity questionnaire
**HR**	Heart rate
**RPM**	Rotations/minute
**Watt**_**max**_	Maximal power
**RPE**	Ratings of perceived exertion
**K**_**2**_ **EDTA**	Potassium ethylene-diamine-tetra-acetic acid
**PBMC**	Peripheral blood mononuclear cell
**D-PBS**	Dulbecco’s phosphate-buffered saline
**RPMI**	Roswell park memorial institute
**FBS**	Fetal bovine serum
**FMO**	Fluorescence minus one
**7-AAD**	7-amino-actinomycin
**EDTA**	Ethylene-diamine-tetra-acetic acid
**ANOVA**	Analysis of variance
**SD**	Standard deviation
**AUC**	Area under the curve
**WBC**	White blood cell

## Data Availability

The original contributions presented in the study are included in the article/Supplementary Material, further inquiries can be directed to the corresponding author.

## References

[B1] Adams G. R. Zaldivar F. P. Nance D. M. Kodesh E. Radom-Aizik S. Cooper D. M. (2011). Exercise and leukocyte interchange among central circulation, lung, spleen, and muscle. Brain Behav. Immun. 25 (4), 658–666. 10.1016/j.bbi.2011.01.002 21238578 PMC4666294

[B2] Agha N. H. Baker F. L. Kunz H. E. Graff R. Azadan R. Dolan C. (2018). Vigorous exercise mobilizes CD34+ hematopoietic stem cells to peripheral blood via the β2-adrenergic receptor. Brain Behav. Immun. 68, 66–75. 10.1016/j.bbi.2017.10.001 29017969 PMC6980177

[B26] Ahmad S. Harris T. Limb E. Kerry S. Victor C. Ekelund U. (2015). Evaluation of reliability and validity of the General Practice Physical Activity Questionnaire (GPPAQ) in 60–74 year old primary care patients. BMC Fam. Pract. 16, 113. 10.1186/s12875-015-0324-8 26329981 PMC4557746

[B3] Allan D. S. Keeney M. Howson-Jan K. Popma J. Weir K. Bhatia M. (2002). Number of viable CD34(+) cells reinfused predicts engraftment in autologous hematopoietic stem cell transplantation. Bone Marrow Transpl. 29, 967–972. 10.1038/sj.bmt.1703575 12098064

[B4] Arroyo E. Tagesen E. C. Hart T. L. Miller B. A. Jajtner A. R. (2022). Comparison of the lymphocyte response to interval exercise versus continuous exercise in recreationally trained men. Brain Behav. Immun. Health 20 (December 2021), 100415. 10.1016/j.bbih.2022.100415 35112091 PMC8790298

[B5] Asfour I. Afify H. Elkourashy S. Ayoub M. Kamal G. Gamal M. (2017). CXCR4 (CD184) expression on stem cell harvest and CD34+ cells post-transplant. Hematology/ Oncol. Stem Cell. Ther. 10 (2), 63–69. 10.1016/j.hemonc.2017.01.002 28282510

[B6] Astori G. Malangone W. Adami V. Risso A. Dorotea L. Falasca E. (2001). A novel protocol that allows short-term stem cell expansion of both committed and pluripotent hematopoietic progenitor cells suitable for clinical use. Blood Cells Mol. Dis. 27 (4), 715–724. 10.1006/bcmd.2001.0439 11778655

[B7] Baker J. M. Nederveen J. P. Parise G. (2017). Aerobic exercise in humans mobilizes HSCs in an intensity-dependent manner. J. Appl. Physiol. 122 (1), 182–190. 10.1152/japplphysiol.00696.2016 27881669 PMC5283849

[B8] Barra N. G. Fan I. Y. Gillen J. B. Chew M. Marcinko K. Steinberg G. R. (2017). High intensity interval training increases natural killer cell number and function in obese breast cancer-challenged mice and obese women. J. Cancer Prev. 22 (4), 260–266. 10.15430/JCP.2017.22.4.260 29302585 PMC5751845

[B9] Basquiera A. L. Abichain P. Damonte J. C. Ricchi B. Sturich A. G. Palazzo E. D. (2006). The number of CD34+ cells in peripheral blood as a predictor of the CD34+ yield in patients going to autologous stem cell transplantation. J. Clin. Apher. 21 (2), 92–95. 10.1002/jca.20062 16106446

[B10] Baumann F. T. Zopf E. M. Nykamp E. Kraut L. Schüle K. Elter T. (2011). Physical activity for patients undergoing an allogeneic hematopoietic stem cell transplantation: benefits of a moderate exercise intervention. Eur. J. Haematol. 87, 148–156. 10.1111/j.1600-0609.2011.01640.x 21545527

[B11] Bay M. L. Heywood S. Wedell-Neergaard A. S. Schauer T. Lehrskov L. L. Christensen R. H. (2020). Human immune cell mobilization during exercise: effect of IL-6 receptor blockade. Exp. Physiol. 105 (12), 2086–2098. 10.1113/EP088864 33006190

[B12] Bellucci R. Propris D. Buccisano F. Lisci A. Leone G. Tabilio A. (1999). Modulation of VLA-4 and L-selectin expression on normal CD34 + cells during mobilization with G-CSF. Bone Marrow Transplant. 23, 1–8. 10.1038/sj.bmt.1701522 10037043

[B13] Bonig H. Papayannopoulou T. (2012). Mobilization of hematopoietic stem/progenitor cells: general principles and molecular mechanisms. Methods Mol. Biol. 904, 1–14. 10.1007/978-1-61779-943-3_1 22890918 PMC3676430

[B14] Bonsignore M. R. Morici G. Riccioni R. Huertas A. Petrucci E. Veca M. (2010). Hemopoietic and angiogenetic progenitors in healthy athletes: different responses to endurance and maximal exercise. J. Appl. Physiol. 109 (1), 60–67. 10.1152/japplphysiol.01344.2009 20448032

[B15] Brevetti G. De Caterina M. Martone V. D. Ungaro B. Corrado F. Silvestro A. (2001). Exercise increases soluble adhesion molecules ICAM-1 and VCAM-1 in patients with intermittent claudication. Clin. Hemorheol. Microcirc. 24 (3), 193–199. 11455059

[B16] Brzoska E. Kowalewska M. Markowska-Zagrajek A. Kowalski K. Archacka K. Zimowska M. (2012). Sdf-1 (CXCL12) improves skeletal muscle regeneration via the mobilisation of Cxcr4 and CD34 expressing cells. Biol. Cell. 104 (12), 722–737. 10.1111/boc.201200022 22978573

[B17] Carney C. E. Buysse D. J. Ancoli-Israel S. Edinger J. D. Krystal A. D. Lichstein K. L. (2012). The consensus sleep diary: standardizing prospective sleep self-monitoring. Sleep 35 (2), 287–302. 10.5665/sleep.1642 22294820 PMC3250369

[B18] Carreras E. Dufour C. Mohty M. Kröger N. (2018). The EBMT Handbook: hematopoietic stem cell transplantation and cellular therapies. EBMT Handb. Hematop. Stem Cell. Transplant. Cell. Ther., 1–702. 10.1007/978-3-030-02278-5 32091673

[B19] Cohen J. (1988). Statistical power analysis for the behavioral Sciences. 2nd Edition.

[B20] Dill D. B. Costill D. L. (1974). Calculation of percentage changes in volumes of blood, plasma, and red cells in dehydration. J. Appl. Physiol. 37 (2), 247–248. 10.1152/jappl.1974.37.2.247 4850854

[B21] Duggan P. R. Guo D. Luider J. Auer I. Klassen J. Chaudhry A. (2000). Predictive factors for long-term engraftment of autologous blood stem cells. Bone Marrow Transplant. 26, 1299–1304. 10.1038/sj.bmt.1702708 11223969

[B22] Emmons R. Niemiro G. M. De Lisio M. (2016b). Exercise as an adjuvant therapy for hematopoietic stem cell mobilization. Stem Cells Int. 2016, 7131359. 10.1155/2016/7131359 27123008 PMC4830735

[B23] Emmons R. Niemiro G. M. Owolabi O. De Lisio M. (2016a). Acute exercise mobilizes hematopoietic stem and progenitor cells and alters the mesenchymal stromal cell secretome. J. Appl. Physiol. 120 (6), 624–632. 10.1152/japplphysiol.00925.2015 26744505

[B24] Ettema G. Lorås H. W. (2009). Efficiency in cycling: a review. Eur. J. Appl. Physiology 106, 1–14. 10.1007/s00421-009-1008-7 19229554

[B25] Felker S. Shrestha A. Bailey J. Pillis D. M. Siniard D. Malik P. (2022). Differential CXCR4 expression on hematopoietic progenitor cells versus stem cells directs homing and engraftment. JCI Insight 7 (9), e151917. 10.1172/jci.insight.151847 PMC9090236 35531956

[B27] Gagnon R. C. Peterson J. J. (1998). Estimation of confidence intervals for area under the curve from destructively obtained pharmacokinetic data. J. Pharmacokinet. Biopharm. 26 (1), 87–102. 10.1023/a:1023228925137 9773394

[B28] Giralt S. Costa L. Schriber J. DiPersio J. Maziarz R. McCarty J. (2014). Optimizing autologous stem cell mobilization strategies to improve patient outcomes: consensus guidelines and recommendations. Biol. Blood Marrow Transplant. 20 (3), 295–308. 10.1016/j.bbmt.2013.10.013 24141007

[B29] Graff R. M. Kunz H. E. Agha N. H. Baker F. L. Laughlin M. Bigley A. B. (2018). β2-Adrenergic receptor signaling mediates the preferential mobilization of differentiated subsets of CD8+ T-cells, NK-cells and non-classical monocytes in response to acute exercise in humans. Brain Behav. Immun. 74, 143–153. 10.1016/j.bbi.2018.08.017 30172948 PMC12977291

[B30] Gratama J. W. Kraan J. Keeney M. Sutherland D. R. Granger V. Barnett D. (2003). Validation of the single-platform ISHAGE method for CD34+ hematopoietic stem and progenitor cell enumeration in an international multicenter study. Cytotherapy. 5 (1), 55–65. 10.1080/14653240310000083 12745591

[B31] Hardy C. J. Rejeski W. J. (2016). Not what, but how one feels: the measurement of affect during exercise. J. Sport Exerc Psychol. 11 (3), 304–317. 10.1123/jsep.11.3.304

[B32] Henig I. Zuckerman T. (2014). Hematopoietic stem cell transplantation—50 Years of evolution and future perspectives. Rambam Maimonides Med. J. 5 (4), e0028. 10.5041/RMMJ.10162 25386344 PMC4222417

[B33] Hénon P. H. Sovalat H. Bourderont D. (2001). Importance of CD34+ cell subsets in autologous PBSC transplantation: the mulhouse experience using CD34+CD38-cells as predictive tool for hematopoietic engraftment. J. Biol. Regul. Homeost. Agents. 15 (1), 62–67. 11388746

[B34] Keeney M. Chin-Yee I. Weir K. Popma J. Nayar R. Robert Sutherland D. (1998). Single platform flow cytometric absolute CD34+ cell counts based on the ISHAGE guidelines. Commun. Clin. Cytom. 34 (2), 61–70. 10.1002/(sici)1097-0320(19980415)34:2<61::aid-cyto1>3.3.co;2-6 9579602

[B35] Krüger K. Pilat C. Schild M. Lindner N. Frech T. Muders K. (2015). Progenitor cell mobilization after exercise is related to systemic levels of G-CSF and muscle damage. Scand. J. Med. Sci. Sports 25 (3), e283–e291. 10.1111/sms.12320 25264280

[B36] Maggs L. Kinsella F. Tracey Chan Y. L. Eldershaw S. Murray D. Nunnick J. (2017). The number of CD56dim NK cells in the graft has a major impact on risk of disease relapse following allo-HSCT. Blood Adv. 1 (19), 1589–1597. 10.1182/bloodadvances.2017008631 29296800 PMC5728471

[B37] Matomäki P. Kainulainen H. Kyröläinen H. (2018). Corrected whole blood biomarkers – the equation of Dill and Costill revisited. Physiol. Rep. 6 (12), e13753. 10.14814/phy2.13749 29939499 PMC6016620

[B38] Miles M. P. Leach S. K. Kraemer W. J. Dohi K. Bush J. A. Mastro A. M. (1998). Leukocyte adhesion molecule expression during intense resistance exercise. J. Appl. Physiol. 84 (5), 1604–1609. 10.1152/jappl.1998.84.5.1604 9572805

[B39] Nederveen J. P. Baker J. Ibrahim G. Ivankovic V. Percival M. E. Parise G. (2020). Hematopoietic stem and progenitor cell (HSPC) mobilization responses to different exercise intensities in young and older adults. J. Sci. Sport Exerc. 2 (1), 47–58. 10.1007/s42978-019-00050-4

[B40] Niemiro G. M. Parel J. Beals J. Van Vliet S. Paluska S. A. Moore D. R. (2017). Kinetics of circulating progenitor cell mobilization during submaximal exercise. J. Appl. Physiol. 122 (3), 675–682. 10.1152/japplphysiol.00936.2016 28082336

[B41] O’Carroll L. Wardrop B. Murphy R. P. Ross M. D. Harrison M. (2019). Circulating angiogenic cell response to sprint interval and continuous exercise. Eur. J. Appl. Physiol. 119 (3), 743–752. 10.1007/s00421-018-04065-7 30673849

[B42] Okutsu M. Ishii K. Kai J. N. Nagatomi R. (2005). Cortisol-induced CXCR4 augmentation mobilizes T lymphocytes after acute physical stress. Am. J. Physiol. Regul. Integr. Comp. Physiol. 288 (3), 591–599. 10.1152/ajpregu.00438.2004 15528395

[B43] Okutsu M. Ishii K. Niu K. Nagatomi R. (2014). Cortisol is not the primary mediator for augmented CXCR4 expression on natural killer cells after acute exercise. J. Appl. Physiol. 117 (3), 199–204. 10.1152/japplphysiol.00176.2014 24947029

[B44] Panch S. R. Szymanski J. Savani B. N. Stroncek D. F. (2017). Sources of hematopoietic stem and progenitor cells and methods to optimize yields for clinical cell therapy. Biol. Blood Marrow Transplant. 23 (8), 1241–1249. 10.1016/j.bbmt.2017.05.003 28495640

[B45] Porrata L. F. Gertz M. A. Geyer S. M. Litzow M. R. Gastineau D. A. Moore S. B. (2004). The dose of infused lymphocytes in the autograft directly correlates with clinical outcome after autologous peripheral blood hematopoietic stem cell transplantation in multiple myeloma. Leukemia 18 (6), 1085–1092. 10.1038/sj.leu.2403341 15042106

[B46] Potocnik A. J. Brakebusch C. Fässler R. (2000). Fetal and adult hematopoietic stem cells require beta1 integrin function for colonizing fetal liver, spleen, and bone marrow. Immunity 12 (6), 653–663. 10.1016/s1074-7613(00)80216-2 10894165

[B47] Robert B. Brown E. B. (1982). Psychophysical bases of perceived exertion. Med. Sci. Sports Exerc 14, 377–381. 10.1249/00005768-198205000-00012 7154893

[B48] Schmid M. Kröpfl J. M. Spengler C. M. (2021b). Correction to: changes in circulating stem and progenitor cell numbers following acute exercise in healthy human subjects: a systematic review and meta-analysis. Stem Cell. Rev. Rep. 17 (4), 1511. 10.1007/s12015-021-10161-7 33825110 PMC8316225

[B49] Schmid M. Martins H. C. Schratt G. Kröpfl J. M. Spengler C. M. (2021a). MiRNA126 – RGS16 – CXCL12 cascade as a potential mechanism of acute exercise-induced precursor cell mobilization. Front. Physiol. 12 (December), 780666. 10.3389/fphys.2021.780666 34955891 PMC8696198

[B50] Sezer O. Possinger K. Metzner B. Illiger H. J. Wattad M. Fuss W. H. H. (2000). Optimal CD34 + cell dose in autologous peripheral-blood stem-cell transplantation. J. Clin. Oncol. 18 (18), 3319–3320. 10.1200/JCO.2000.18.18.3319 10986066

[B51] Simpson R. J. Bigley A. B. Agha N. Hanley P. J. Bollard C. M. (2017). Mobilizing immune cells with exercise for cancer immunotherapy. Exerc Sport Sci. Rev. 45 (3), 163–172. 10.1249/JES.0000000000000114 28418996 PMC6814300

[B52] Simpson R. J. Boßlau T. K. Weyh C. Niemiro G. M. Batatinha H. Smith K. A. (2021). Exercise and adrenergic regulation of immunity. Brain Behav. Immun. 97 (June), 303–318. 10.1016/j.bbi.2021.07.010 34302965

[B53] Simpson R. J. Kunz H. Agha N. Graff R. (2015). Exercise and the regulation of immune functions. Prog. Mol. Biol. Transl. Sci. 135, 355–380. 10.1016/bs.pmbts.2015.08.001 26477922

[B54] Spielberger C. D. Gonzalez-Reigosa F. Martinez-Urrutia A. Natalicio L. F. S. Natalicio D. S. (2017). The state-trait anxiety inventory. Revista Interamericana de Psicología/Interamerican J. Psychol. 5 (3 & amp). 4 SE-Articles).

[B55] Stroncek D. F. Clay M. E. Herr G. Smith J. Jaszcz W. B. Ilstrup S. (1997). The kinetics of G-CSF mobilization of CD34+ cells in healthy people. Transfus. Med. 7 (1), 19–24. 10.1046/j.1365-3148.1997.d01-75.x 9089980

[B56] Sutherland D. R. Anderson L. Keeney M. Nayar R. Chin-Yee I. (1996). The ISHAGE guidelines for CD34+ cell determination by flow cytometry. International Society of Hematotherapy and Graft Engineering. J. Hematother Stem Cell. Res. 5 (3), 213–226. 10.1089/scd.1.1996.5.213 8817388

[B57] Taylor G. S. Shaw A. Smith K. Capper T. E. Scragg J. H. Cronin M. (2021). Type 1 diabetes patients increase CXCR4+ and CXCR7+ haematopoietic and endothelial progenitor cells with exercise, but the response is attenuated. Sci. Rep. 11 (1), 14502. 10.1038/s41598-021-93886-2 34267242 PMC8282661

[B58] Taylor J. L. Holland D. J. Spathis J. G. Beetham K. S. Wisløff U. Keating S. E. (2019). Guidelines for the delivery and monitoring of high intensity interval training in clinical populations. Prog. Cardiovasc Dis. 62 (2), 140–146. 10.1016/j.pcad.2019.01.004 30685470

[B59] Turner J. E. Wadley A. J. Aldred S. Fisher J. P. Bosch J. A. Campbell J. P. (2016). Intensive exercise does not preferentially mobilize skin-homing T cells and NK cells. Med. Sci. Sports Exerc 48 (7), 1285–1293. 10.1249/MSS.0000000000000914 26918560

[B60] Walsh N. P. Gleeson M. Shephard R. J. Jeffrey M. G. Woods A. Bishop N. C. (2011). Position statement. Part one: immune function and exercise. Exerc Immunol. Rev. 17, 6–63. 21446352

[B61] Weston K. S. Wisløff U. Coombes J. S. (2014). High-intensity interval training in patients with lifestyle-induced cardiometabolic disease: a systematic review and meta-analysis. Br. J. Sports Med. 48 (16), 1227–1234. 10.1136/bjsports-2013-092576 24144531

