# Supplementary material for: Brief cycling intervals incrementally increase the number of hematopoietic stem and progenitor cells in human peripheral blood
Source: Front Physiol. 2024 Jul 30;15:1327269. doi: 10.3389/fphys.2024.1327269 (PMC11319260; doi:10.3389/fphys.2024.1327269)
Supplement: Supplementary file 1 [file DataSheet1.docx]

Supplementary Material

# Supplementary Tables

# Supplementary Table 1. Statistical output of changes in peripheral blood concentrations of all immune cell subsets between Pre- and Post-Ex in all trials. The ‘Interval 4 (30 minutes)’ timepoint is represented as ‘Post-Ex’.

| **Cell Subset** | **Timepoint** | | **Cycling Trial** | | | **P Value** | | |  |
| --- | --- | --- | --- | --- | --- | --- | --- | --- | --- |
|  |  |  | **MICE** | **HV-HIIE** | **LV-HIIE** |  |  |  |  |
| **WBC** | Pre | | 5620 ± 1273^*^ | 5457 ± 1112^*^ | 5520 ± 1491^*^ | < 0.0001 | | |  |
|  | Post-Ex | | 6966 ± 1801^*^ | 8078 ± 1840^*^ | 8781 ± 2581^*^ |  |  |  |  |
| **Neutrophils** | Pre | | 2958 ± 695^*^ | 2912 ± 584^*^ | 3304 ± 1857^*^ | 0.05 | | |  |
|  | Post-Ex | | 3807 ± 1227^*^ | 4171 ± 1123^*^ | 4683 ± 2150^*^ |  |  |  |  |
| **Lymphocytes** | Pre | | 1882 ± 533^*^ | 1864 ± 587^*^ | 1847 ± 580^*^ | < 0.0001 | | |  |
|  | Post-Ex | | 2315 ± 630^2,*^ | 3085 ± 1015^*^ | 3344 ± 1360^2,*^ |  |  |  |  |
| **Monocytes** | Pre | | 445 ± 105 | 416 ± 106 | 1790 ± 2629^*^ | 0.01 | | |  |
|  | Post-Ex | | 619 ± 167^2^ | 687 ± 204^3^ | 2547 ± 3336^2,3,*^ |  |  |  |  |
| **T cells** | Pre | | 1261 ± 417 | 1238 ± 434^*^ | 1187 ± 355^*^ | 0.03 | | |  |
|  | Post-Ex | | 1388 ± 441 | 1630 ± 678^*^ | 1715 ± 719^*^ |  |  |  |  |
| **CD56^dim^ NK** | Pre | | 197 ± 87^*^ | 202 ± 85^*^ | 222 ± 88^*^ | 0.0002 | | |  |
|  | Post-Ex | | 433 ± 132^1,2,*^ | 817 ± 227^1,*^ | 943 ± 442^2,*^ |  |  |  |  |
| **CD56^bright^ NK** | Pre | | 8.64 ± 3.62 | 7.38 ± 3.95^*^ | 8.05 ± 3.62^*^ | 0.02 | | |  |
|  | Post-Ex | | 10.33 ± 4.59 | 14.31 ± 7.20^*^ | 15.84 ± 9.23^*^ |  |  |  |  |
| **HSPCs** | Pre | | 2.15 ± 0.90 | 2.05 ± 0.86 | 1.84 ± 1.55^*^ | < 0.0001 | | |  |
|  | Post-Ex | | 1.76 ± 0.82^2^ | 2.42 ± 1.04 | 3.21 ± 2.00^2,*^ |  |  |  |  |
| Data displayed as mean ± SD. | | | | | | |  | | |
| ^1^, significant difference between MICE and HV-HIIE (P < 0.05) | | | | | | |  | | |
| ^2^, significant difference between MICE and LV-HIIE (P < 0.05) | | | | | | |  | | |
| ^3^, significant difference between HV-HIIE and LV-HIIE (P < 0.05) | | | | | | |  | | |
| ^*^, significant difference between Pre- and Post-Ex (P < 0.05) | | | | | | |  | | |
|  | |  |  |  |  |  |  |  |  |
| Abbreviations: MICE, moderate intensity continuous exercise; HV-HIIE, high volume-high intensity interval exercise; LV-HIIE, low volume-high intensity interval exercise; WBC, white blood cell; NK, natural killer and HSPCs, haematopoietic stem and progenitor cells.  The ‘Interval 4 (30 minutes)’ timepoint is represented as ‘Post-Ex’. | | | | | | | |  |  |

**Supplementary Table 2:** Sleep efficiency and state and trait anxiety prior to each experimental trial.

| **Cycling Trial** | | | | |
| --- | --- | --- | --- | --- |
| **Parameter** | **MICE** | **HV-HIIE** | **LV-HIIE** | **P-Value** |
| Anxiety State (S_anxiety_) | 45.18 ± 3.49 | 44.91 ± 3.83 | 44.55 ± 3.80 | > 0.05 |
| Anxiety Trait (T_anxiety_) | 44.36 ± 3.61 | 45.27 ± 4.32 | 44.09 ± 3.70 | > 0.05 |
| Sleep Efficiency (%) | 87.36 ± 8.36 | 86.84 ± 10.50 | 86.27 ± 8.14 | > 0.05 |
| Data displayed as mean ± SD.  P > 0.05 indicates no signiﬁcant differences between trials. A repeated measures ANOVA revealed no significant differences in state (F (2, 30) = 0.08, P = 0.92) and trait (F (2, 30) = 0.28, P = 0.76) anxiety levels or sleep efficiency (F (2, 30) = 0.04, P = 0.96) before undertaking the three experimental trials.  Abbreviations: MICE, moderate intensity continuous exercise; HV-HIIE, high volume-high intensity interval exercise; LV-HIIE, low volume-high intensity interval exercise. | | | | |

**Supplementary Figures**

**
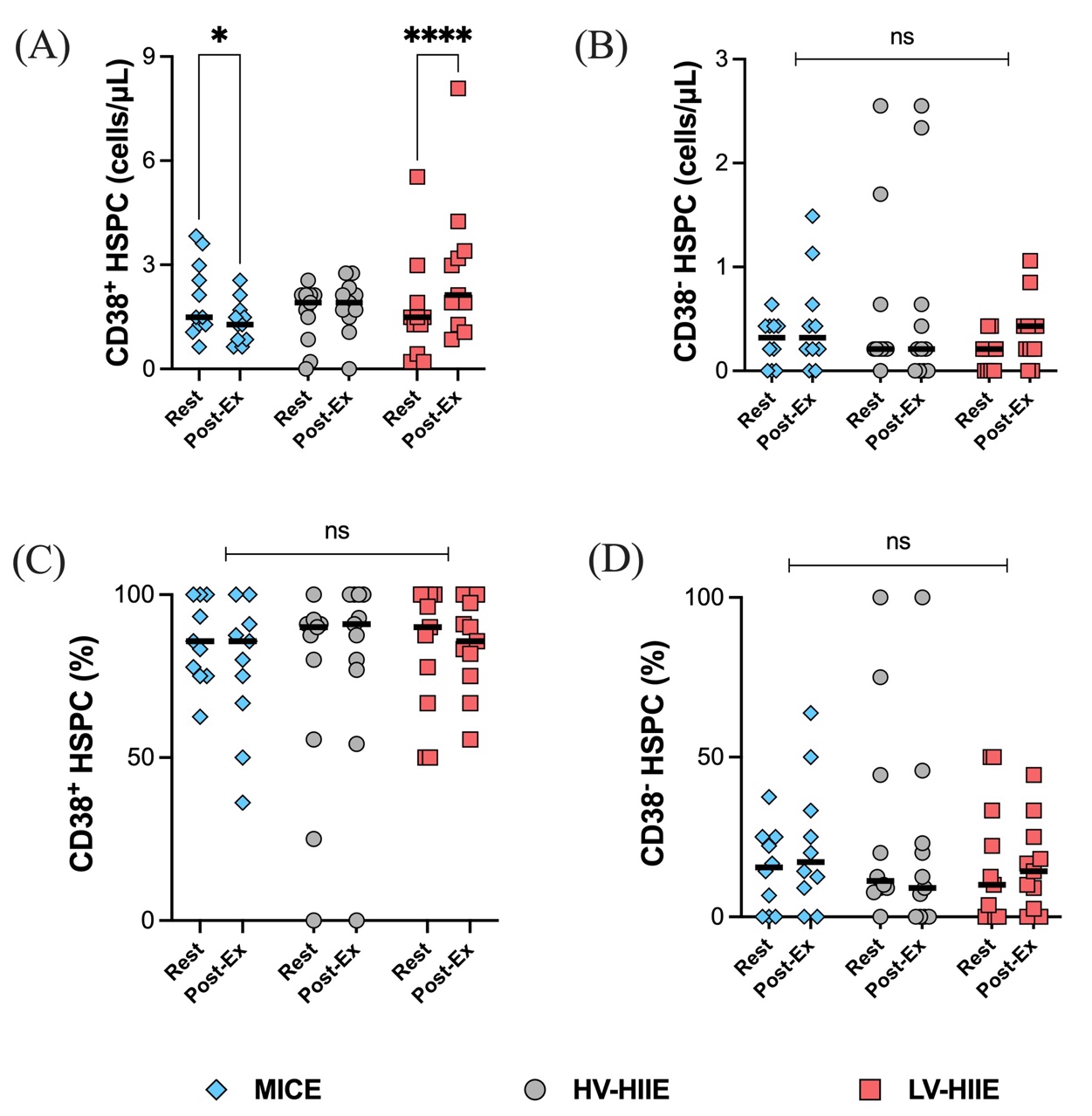
**

**Supplementary Figure 1.** Changes in the peripheral blood concentration (**A, B**) and frequency (**C, D**) of CD38^+^ and CD38^-^ HSPCs between rest and Interval 4 (30 minutes) in MICE (blue bars), HV-HIIE (grey bars) and LV-HIIE (red bars). Data was obtained using SPFC analysis. Values are means ± SD. * indicates signiﬁcant differences between Pre- and Post-Ex. *P < 0.05, ****P < 0.0001. Ns indicates no signiﬁcant differences between timepoints or trials: P > 0.05. The ‘Interval 4 (30 minutes)’ timepoint is represented as ‘Post-Ex’.

# Supplementary Figure 2. A statistical comparison of the fold change (Interval 4 (30 minutes) vs. rest) between cell subsets (White blood cells (WBC), neutrophils, lymphocytes, monocytes, T cells, CD56^dim^ NK cells, CD56^bright^ NK cells and HSPCs) in MICE (A), HV-HIIE (B) and LV-HIIE (C). The grey area represents no comparison between row vs. column and vice versa. White boxes and # represent significant differences between table row vs. column. Red boxes and * represent significant differences between table column vs. row. Blue boxes and ns indicate no significant differences between subsets. *P < 0.05, **P < 0.01, ***P < 0.001, ##P < 0.01, ###P < 0.001.


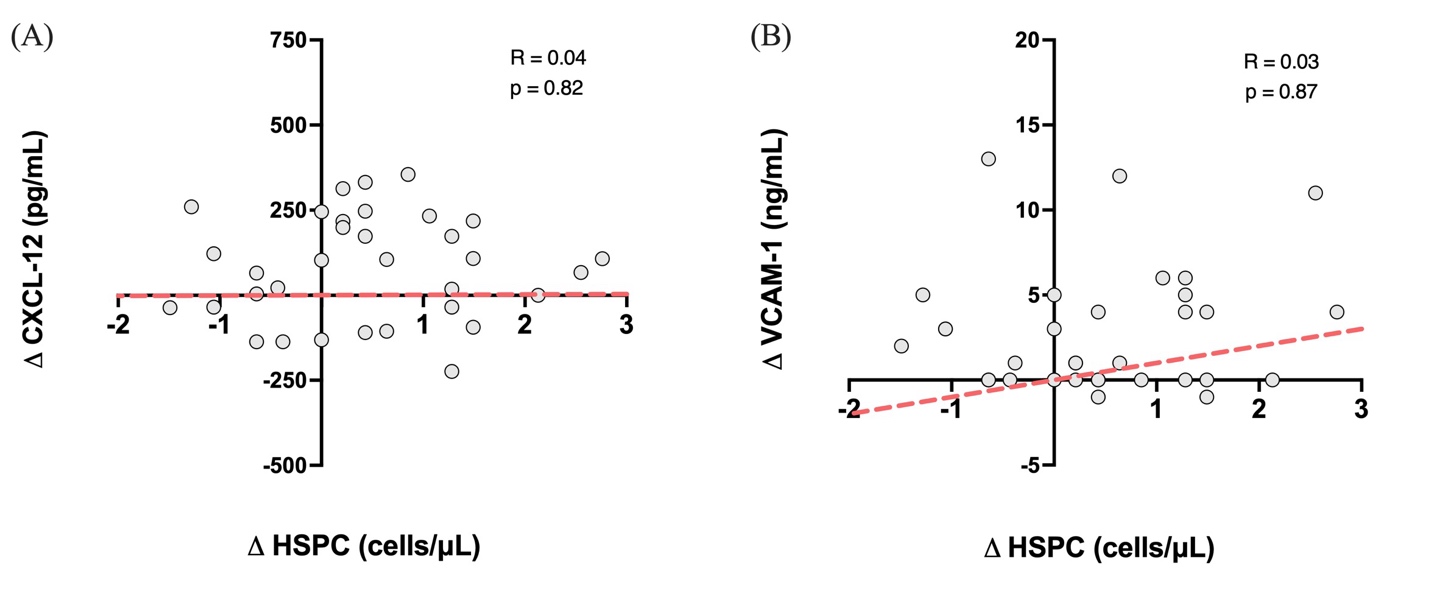


# Supplementary Figure 3. The relationship between HSPC mobilisation and changes in plasma chemokine concentrations between rest and Interval 4 (30 minutes) in all trials. The delta change (∆) of HSPC concentration was plotted against ∆ CXCL-12 (A) and ∆ VCAM-1 (B), and Pearson and Spearman correlation coefficients determined respectively.
